# Phomopsichin A–D; Four New Chromone Derivatives from Mangrove Endophytic Fungus *Phomopsis* sp. 33#

**DOI:** 10.3390/md14110215

**Published:** 2016-11-22

**Authors:** Meixiang Huang, Jing Li, Lan Liu, Sheng Yin, Jun Wang, Yongcheng Lin

**Affiliations:** 1 School of Pharmaceutical Sciences, Sun Yat-sen University, Guangzhou 510006, China; huangmx7@mail2.sysu.edu.cn (M.H.); yinsh2@mail.sysu.edu.cn (S.Y.); 2 School of Marine Sciences, Sun Yat-sen University, Guangzhou 510006, China; lijing356@mail.sysu.edu.cn; 3 Key Laboratory of Functional Molecules from Oceanic Microorganisms (Sun Yat-sen University), Department of Education of Guangdong Province, Guangzhou 510080, China; 4 South China Sea Bio-Resource Exploitation and Utilization Collaborative Innovation Center, Guangzhou 510006, China; 5 School of Chemistry and Chemical Engineering, Sun Yat-sen University, Guangzhou 510275, China; ceslyc@mail.sysu.edu.cn

**Keywords:** mangrove endophytic fungi, *Phomopsis* sp., secondary metabolites, chromone derivatives

## Abstract

Four new chromone derivatives, phomopsichins A–D (**1**–**4**), along with a known compound, phomoxanthone A (**5**), were isolated from the fermentation products of mangrove endophytic fungus *Phomopsis* sp. 33#. Their structures were elucidated based on comprehensive spectroscopic analysis coupled with single-crystal X-ray diffraction or theoretical calculations of electronic circular dichroism (ECD). They feature a tricyclic framework, in which a dihydropyran ring is fused with the chromone ring. Compounds **1**–**5** showed weak inhibitory activities on acetylcholinesterase as well as α-glucosidase, weak radical scavenging effects on 1,1-diphenyl-2-picrylhydrazyl (DPPH) as well as OH, and weak antimicrobial activities. Compounds **1**–**4** showed no cytotoxic activity against MDA-MB-435 breast cancer cells. Their other bioactivities are worthy of further study, considering their unique molecular structures.

## 1. Introduction

**Figure 1:**
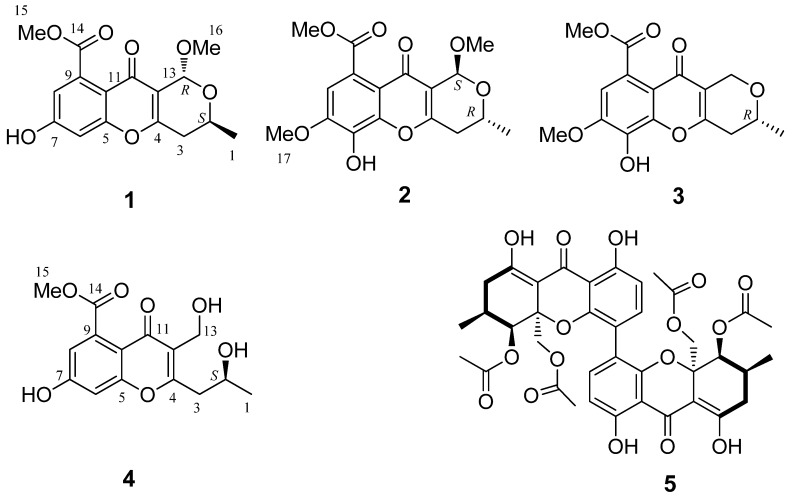
The chemical structures of compounds **1**–**5**.

The chromone family of natural products exhibit a range of biological activities including anticancer, anti-inflammatory, antibacterial, antiviral, atypical antipsychotic, and anti-platelet properties [1,2,3,4,5,6,7,8,9]. In our continuous investigation of new bioactive secondary metabolites from the mangrove endophytic fungi in the South China Sea, four new chromone derivatives, phomopsichin A–D (**1**–**4**), along with a known compound, phomoxanthone A (**5**), were isolated from the metabolic products of endophytic fungus *Phomopsis* sp. 33# from the bark of the mangrove plant *Rhizophora stylosa*. Compounds **1**–**3** (Figure 1) featured a tricyclic framework in which a dihydropyran ring is fused at C-3 and C-4 of the chromone ring. To our knowledge, the compounds with this type skeleton number approximately 10, which were reported to exhibit the activity attenuating resistin-induced adhesion of HCT-116 colorectal cancer cells to endothelial cells [10], the activity interrupting the dimer formation of Aβ17–42 peptide associated to Alzheimer’s disease [11], inhibitory activity against metallo-β-lactamases [12], moderate antibacterial activity and weak cytotoxic activity [13,14,15]. In this study, we report the isolation, structural elucidation, and exploration on the biological activities of compounds **1**–**5**.

## 2. Results

### 2.1. Structure Elucidation

**Figure 2:**
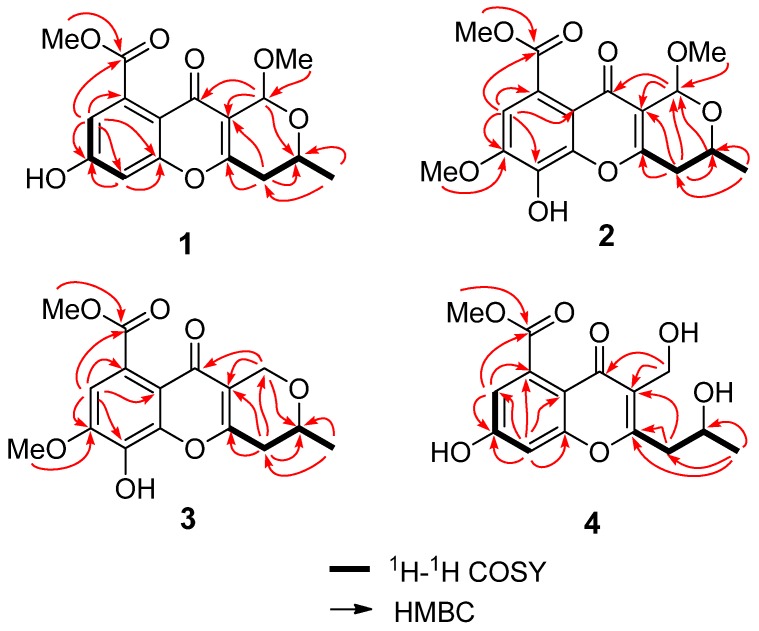
The key ^1^H-^1^H COSY and HMBC correlations of compounds **1**–**4**.

Phomopsichin A (**1**, Figure 1) was obtained as a white solid and had a molecular formula of C_16_H_16_O_7_ as determined by its datum of high resolution electrospray ionization mass spectroscopy (HRESIMS) (observed *m*/*z* 319.08184 M^−^, calculated 319.08233), requiring nine degrees of unsaturation. The ^13^C-NMR and distortionless enhancement by polarization transfer (DEPT) spectra (Table 1) indicated the presence of two carbonyl groups (*δ_c_* 169.5 and 173.2), eight olefinic carbons, two sp^3^ CH groups, one sp^3^ CH_2_ group, two methoxy groups, and one methyl group. The ^1^H-NMR and ^1^H-^1^H correlation spectroscopy (COSY) (Table 1 and Figure 2) showed the signals of two *m*-hydrogens of phenol (*δ*_H_ 6.93 d *J* = 2.4 Hz; 6.85 d *J* = 2.4 Hz), two methoxy groups (*δ*_H_ 3.85/3.42), and one 2-oxo-propyl group (*δ*_H_ 1.34 d *J* = 6.0 Hz; 4.34 m; 2.67 dd *J* = 18.0, 4.0 Hz; 2.58 dd *J* = 18.0, 4.0 Hz). The remaining two degrees of unsaturation supported a tricyclic carbon framework of dihydropyrano[4,3-b]chromen-10(1H)-one in **1**, which was confirmed by the correlations between H-13 and C-2/C-11 in heteronuclear multiple-bond correlation (HMBC) spectroscopy. In the HMBC spectrum (Figure 2), rich correlation data allowed us to unambiguously establish the locations of substituents on the carbon skeleton. The HMBC correlation between H-8 and C-14 revealed that the carbonyl group was located at the C-9 position; the correlation between H_3_-1 and C-3 demonstrated that the CH_3_-1 was located at the C-2 position; and the correlations between H_3_-15 and C-14 as well as between H_3_-16 and C-13 indicated that the two methoxy groups were located at the C-14 and C-13 positions, respectively. One hydroxyl group was identified at the C-7 position based on the lower field chemical shift (*δ_c_* 162.6, C-7).

**Figure 3:**
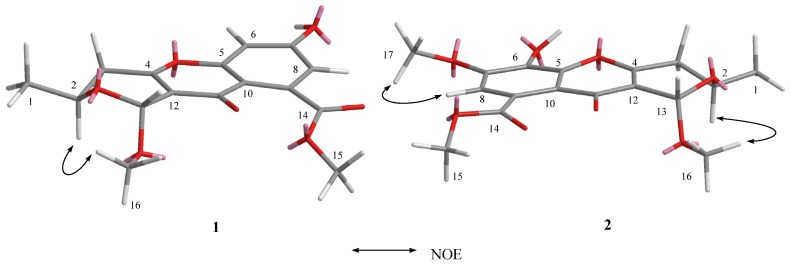
The key correlations of compounds **1** and **2** in NOESY.

The relative stereochemistry of **1** was established by its nuclear Overhauser effect spectroscopy (NOESY). The NOE correlation between H-2 and H_3_-16 indicated the relative stereochemistry of **1** as shown in Figure 3.

**Figure 4:**
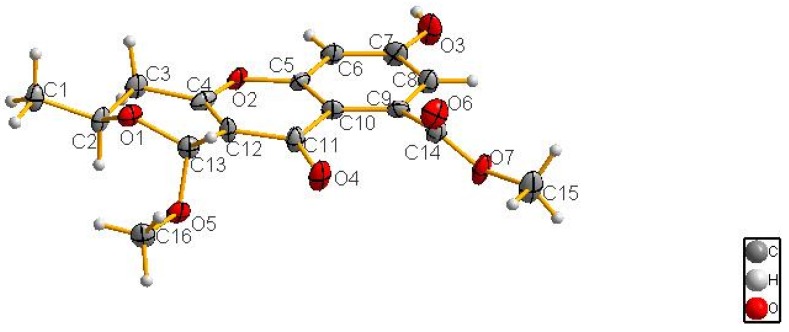
The X-ray single-crystal structure of **1**.

The complete structure and stereochemistry of **1** were further confirmed by X-ray diffraction analysis (Figure 4). The final refinement of the Cu Kα data resulted in a small Flack parameter of 0.02(3), allowing an unambiguous assignment of the absolute configuration of **1** as 2*S*, 13*R* (Figure 1).

Phomopsichin B (**2**, Figure 1) was obtained as a white solid and had a molecular formula of C_17_H_18_O_8_ based on HRESIMS data (observed *m*/*z* 349.09241 M^−^, calculated 349.09289), with one more CH_3_O group than compound **1**. The ^1^H-NMR, ^13^C-NMR, and HMBC spectra of **2** were very similar to those of **1** (Table 1), except for the absence of the H-6 signal, and an added CH_3_O-17 signals (*δ_H_*_/*C*_ 3.98/56.8). The added CH_3_O-17 was located at the C-7 position based on the NOE correlation between H-17 and H-8. One hydroxyl group was identified at the C-6 position based on the chemical shift of C-6 (*δ*_c_ 134.7) as well as the HMBC correlation between H-8 and C-6.

The relative stereochemistry of **2** was established by its NOESY. The NOE correlation between H-2 and H_3_-16, similar to those of **1**, indicated the relative stereochemistry of **2** as shown in Figure 3.

**Figure 5:**
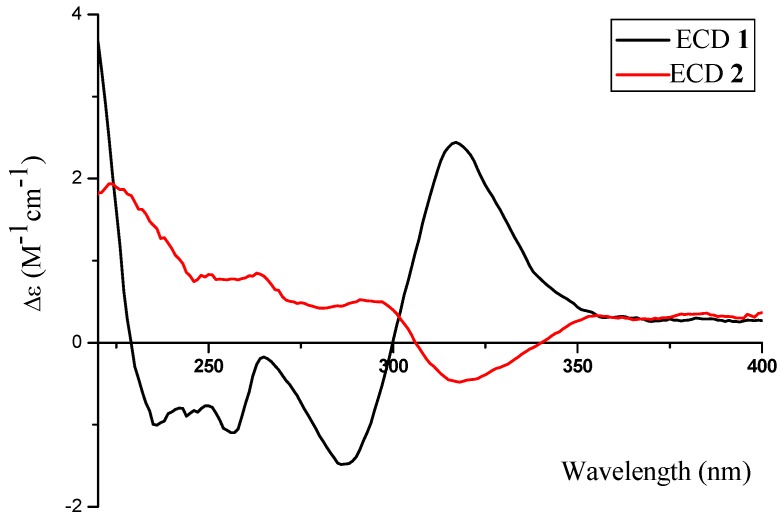
The ECD spectra of **1** and **2**.

Compounds **2** and **1** have identical chiral spheres, just opposite in the signs of their specific rotation data; their ECD spectra were symmetric (Figure 5). The ECD spectrum of **2** showed negative Cotton effect at 318 (*Δε* −0.48) nm as well as positive one at 291 (*Δε* +0.53) nm. Meanwhile, the ECD spectrum of **1** displayed opposite Cotton effects at the same wavelengths. For the above reasons, the absolute configuration of **2** was suggested as 2*R*, 13*S*.

**Figure 6:**
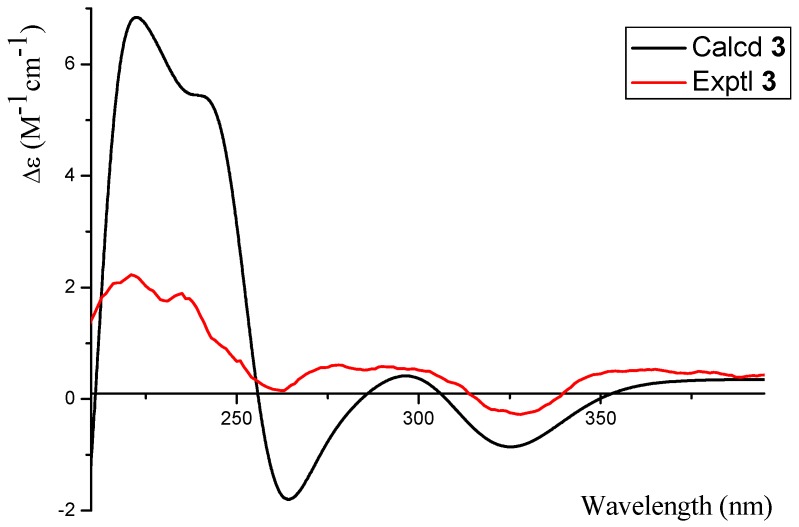
The calculated and experimental ECD spectra of **3**.

Phomopsichin C (**3**, Figure 1) was obtained as a white solid and had a molecular formula of C_16_ H_16_O_7_ based on HRESIMS data (observed *m*/*z* 319.08200 M^−^, calculated 319.08233). The ^1^H-NMR, ^13^C-NMR, ^1^H-^1^H COSY, and HMBC spectra of **3** were very similar to those of compound **2** (Table 1, Figure 2), except for the changes of CH-13 signals (*δ_H_*_/*C*_ 5.57/94.5) in **2** to CH_2_-13 signals (*δ_H_*_/*C*_ 4.82 d; 4.48 d/62.5) in **3**. These results suggested that compound **3** is lacking a methoxy group at the C-13 position. The absolute configuration of compound **3** was determined as 2*R* by the result that the experimental ECD and calculated ECD spectrum for 2*R* isomer matched exactly (Figure 6).

Phomopsichin D (**4**, Figure 1) had a molecular formula of C_15_H_16_O_7_ based on HRESIMS data (observed *m/z* 307.08194 M^−^, calculated 307.08233), requiring eight degrees of unsaturation. The ^1^H-NMR, ^13^C-NMR, ^1^H-^1^H COSY, and HMBC spectra of **4** were very similar to those of **1** (Table 1 and Figure 2), except for the change of CH-13 signals (*δ_H_*_/*C*_ 5.40/95.2) in **1** to CH_2_OH-13 signals (*δ_H_*_/*C*_ 4.55/55.0) and the absence of a methoxy group signal in **4**. A dicyclic 4*H*-chromen-4-one segment of **4** was decided based on its eight degrees of unsaturation, which was supported by the absence of HMBC correlation between H-13 and C-2. The hydroxymethyl group was located at C-12 based on the HMBC correlation between H-13 and C-11. A 2-hydroxypropyl group was located at C-4 based on the HMBC correlations between H-1 and C-4, as well as, between H-3 and C-12.

**Figure 7:**
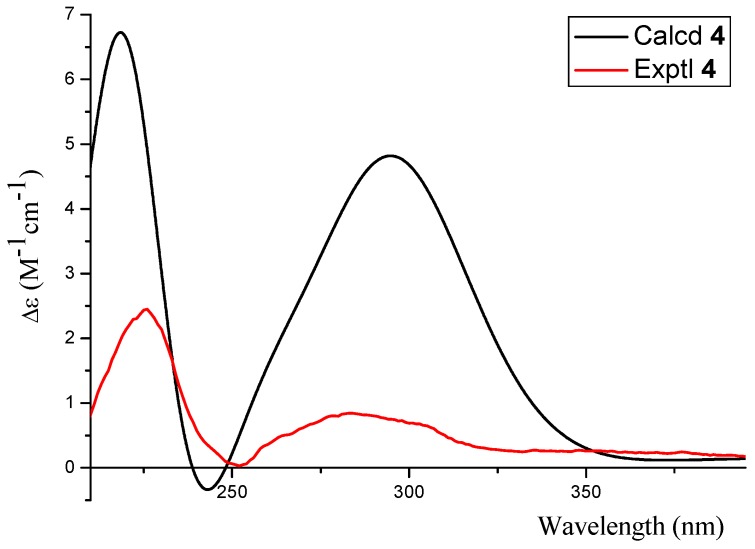
The calculated and experimental ECD spectra of **4**.

The absolute configuration of **4** was confirmed as 2*S* based on the result that the experimental data and calculated ECD spectrum for the 2*S* isomer matched exactly (Figure 7).

Compound **5** was identified as phomoxanthone A (**5**, Figure 1) by comparison of its spectral data with that of the literature [16,17]; both compound **5** and phomoxanthone A had the same NMR, MS, ECD data (Figure S27, in Supplementary Materials) and specific rotation data.

### 2.2. Biological Evaluation

The various bioactivities of compounds **1**–**5** were evaluated in vitro. The five compounds displayed low inhibitory activities on acetylcholinesterase (AchE) as well as α-glucosidase, weak radical scavenging effects on DPPH as well as OH, and low antimicrobial activity against 13 pathogenic bacteria strains (Tables S8 and S9, in Supplementary Materials). Compounds **1**–**4** showed no cytotoxic activity against MDA-MB-435 breast cancer cells. It was reported that phomoxanthone A (**5**) has strong pro-apoptotic activity and immunostimulatory activity [17], so we did not consider its cytotoxicity assays in the study.

## 3. Materials and Methods

### 3.1. General Experimental Procedures

Acetylcholinesterase (AchE), S-acetylthiocholine iodide, 5,5′-dithio-bis-(2-nitrobenzoic acid), huperzine A, α-glucosidase and *p*-nitrophenyl-α-d-glucopyranoside were purchased from Sigma (St. Louis, MO, USA); 1,1-diphenyl-2-picrylhydrazyl (DPPH), H_2_O_2_, 1,10-phenanthroline, FeSO_4_, and other reagents were of analytical grade and commercially available; methanol was of chromatographic grade; potato dextrose agar (PDA) medium was purchased from Beijing L and Bridge Technology Co. Ltd. (Beijing, China).

Optical rotation measurements were carried out using a Bellingham-Stanley 37–440 polarimeter (Bellingham Stanley Ltd., Kent, UK). UV spectra were determined using a UV-240 spectrophotometer (Shimadzu, Tokyo, Japan). ECD spectra were measured using a Chirascan Circular Dichroism Spectrometer (Applied Photophysics, London, UK). IR spectra were measured on a TENSOR37 spectrometer (Bruker Optics, Ettlingen, Germany). The ^1^H-NMR and ^13^C-NMR data were acquired using a Bruker Avance 400 spectrometer at 400 MHz for ^1^H nuclei and 100 MHz for ^13^C nuclei (Bruker Biospin, Rheinstetten, Germany). Tetramethylsilane (TMS) was used as an internal standard, and the chemical shifts (*δ*) were expressed in ppm. The HRESIMS were obtained using a LTQ-Orbitrap LC-MS (Thermo Fisher, Frankfurt, Germany). Single-crystal data were carried out on an Agilent Technologies Gemini A Ultra system (Agilent Tech, Santa Clara, CA, USA). HPLC was performed using a 515 pump with a UV 2487 detector (Waters, Milford, CT, USA) and an Ultimate XB-C-18 column (250 mm × 10 mm, 5 μm; Welch, Maryland, USA). Normal pressure preparative column chromatography was carried out on RP-18 gel (25–40 μm, Daiso Inc., Osaka, Japan), silica gel (200–400 mesh, Qingdao Marine Chemical Inc., Qingdao, China), or a Sephadex-LH-20 (GE Healthcare, Stockholm, Sweden) for reverse and direct phase elution modes, respectively. The thin-layer chromatography was performed over F_254_ glass plates (Qingdao Marine Chemical Inc.) and analyzed under UV light (254 and 366 nm).

### 3.2. Fungal Material

Endophytic fungus *Phomopsis* sp. 33# was isolated with PDA medium from the bark of the mangrove plant *Rhizophora stylosa*, collected in the intertidal region of Zhanjiang, in Guangdong Province, China, and identified according to its morphological characteristics and internal transcribed spacer (ITS) region [18]. A voucher specimen is deposited in our laboratory at −20 °C.

### 3.3. Fermentation, Extraction, and Isolation

Small agar slices bearing mycelia were placed in 1000 mL Erlenmeyer flasks containing rice medium (composed of 60 g rice, 80 mL distilled water, and 0.24 g sea salt) and incubated for 30 days at 28 °C. In total, 140 flasks of culture were obtained. Cultures were extracted with EtOAc. In total, 250 g crude extract was obtained by evaporation of EtOAc. The crude extract was suspended in H_2_O (3 L) and partitioned with *n*-hexane (5 L × 2) and EtOAc (5 L × 2) to give *n*-hexane (90 g) and EtOAc (110 g) extracts, respectively.

The EtOAc extract was subjected to a silica gel column, eluted with a *n*-hexane-EtOAc gradient (from 100:0 to 0:100) to obtain six fractions (Fractions 1–6). Fraction 2 (15 g) was extracted with 300 mL of chloroform to give dark yellow liquid phases and solid. The chloroform-soluble fraction was evaporated to dryness and washed with methanol (50 mL × 6) to give compound **5** (a light yellow solid, 5.2 g). Fraction 4 (15 g) was chromatographed over a column of RP-18 gel (2.5 cm × 30 cm, MeOH-H_2_O gradient from: 100:0 to 40:60) to obtain five fractions (Fractions 4.1–4.5). Fraction 4.1 and 4.3–4.5 were separated by HPLC (MeOH-H_2_O, 20:80, 2 mL/min, 254 nm), respectively, and then were purified separately using a Sephadex LH-20 column (MeOH) until pure compound **1** (17 mg), compound **2** (17mg), compound **3** (10 mg), and compound **4** (22 mg) were obtained.

### 3.4. Spectral Data

Phomopsichin A (**1**): white solid; [a]${}_{D}^{25}$ +12.5 (*c* 0.48, MeOH); UV (MeOH) λ_max_ (log*ε*) 290 (4.0), 218 (4.4) nm; ECD (MeOH) λ_max_ (*Δε*) 312 (+2.4), 281 (−1.5), 231 (−1.0) nm; IR (KBr) ν_max_ 3410, 2925, 1655 cm^−1^; for ^1^H-NMR and ^13^C-NMR data, see Table 1; ESIMS *m*/*z* 319.0 [M^−^]; HRESIMS *m*/*z* 319.08184 [M^−^] (calculated for C_16_H_15_O_7_, 319.08233).

Crystal structure determination of phomopsichin A (**1**): crystal data for C_16_H_16_O_7_ (*M* = 320.29 g/mol): monoclinic, space group P2_1_ (no. 4), *a* = 7.6234 (9) Å, *b* = 15.5426 (17) Å, *c* = 12.4889 (13) Å, *β* = 96.241 (10)°, *V* = 1471.0 (3) Å^3^, *Z* = 4, *T* = 150 (2) K, μ (CuKα) = 0.973 mm^−1^, *D*calc = 1.446 g/cm^3^, 15,309 reflections measured (7.12° ≤ 2Θ ≤ 133.92°), 5018 unique (*R*_int_ = 0.0877, R_sigma_ = N/A) which were used in all calculations. The final *R*_1_ was 0.0849 (>2sigma(I)) and *wR*_2_ was 0.2334 (all data). The crystallographic data of **1** have been deposited at the Cambridge Crystallographic Data Centre (CCDC), CCDC Depository Request CRM: 0001000638303.

Phomopsichin B (**2**): white solid; [a]${}_{D}^{25}$ −8.0 (*c* 0.89, MeOH); UV (MeOH) λ_max_ (log*ε*) 299 (3.8), 238 (4.4) nm; ECD (MeOH) λ_max_ (*Δε*) 318 (−0.48), 291 (+0.53) nm; IR (KBr) ν_max_ 3284, 2922, 1649 cm^−1^; for ^1^H-NMR and ^13^C-NMR data, see Table 1; ESIMS *m*/*z* 349.0 [M^−^]; HRESIMS *m*/*z* 349.09241 [M^−^] (calculated for C_17_H_17_O_8_, 349.09289).

Phomopsichin C (**3**): white solid; [a]${}_{D}^{25}$ −72 (*c* 0.05, MeOH); UV (MeOH) λ_max_ (log*ε*) 303 (3.9), 239 (4.5) nm; ECD (MeOH) λ_max_ (*Δε*) 328 (−0.27), 278 (+0.61), 221 (+2.2) nm; IR (KBr) ν_max_ 3325, 2922, 1665 cm^−1^; for ^1^H-NMR and ^13^C-NMR data, see Table 1; ESIMS *m/z* 319.1 [M^−^], 206, 175; HRESIMS *m*/*z* 319.08200 [M^−^] (calculated for C_16_H_15_O_7_, 319.08233).

Phomopsichin D (**4**): white solid; [a]${}_{D}^{25}$ +102 (*c* 0.05, MeOH); UV (MeOH) λ_max_ (log*ε*) 293 (3.8), 221 (4.2) nm; ECD (MeOH) λ_max_ (*Δε*) 284 (+0.83), 226 (+2.4) nm; IR (KBr) ν_max_ 3265, 2912, 1707 cm^−1^; for ^1^H-NMR and ^13^C-NMR data, see Table 1; ESIMS *m*/*z* 307.0 [M^−^]; HRESIMS *m*/*z* 307.08194 [M^−^] (calculated for C_15_H_15_O_7_, 307.08233).

### 3.5. Computational Analyses

All of the theoretical methods and the basis set used for optimization and spectrum calculation were recommended in previous studies [19,20]. All of the theoretical calculations, including geometry optimization, frequency analysis, and ECD spectrum prediction, were carried out with the density functional theory (DFT) and time-dependent density functional theory (TDDFT) methods in the Gaussian 09 software package (Gaussian Inc., Wallingford, CT, USA) [21]. The geometry optimizations were performed at the B3LYP/6-31+G (d) level in the gas phase. Based on the final optimized structure, the ECD spectra were calculated at the PBE1PBE-SCRF/6-311++g (d, p) level using the Polarized Continuum Model (PCM) with methanol as a solvent. The theoretical predicted ECD spectra were fitted in the SpecDis 1.6 software package (University of Würzburg, Würzburg, Germany) [22].

### 3.6. X-ray Crystallographic Analysis of Compound ***1***

Single crystals of compound **1** were obtained from CH_3_OH-EtOAc. A suitable crystal was selected and all crystallographic data were collected at 150 K with Cu/K*α* radiation (*λ* = 1.54178 Å). Using Olex2 (OlexSys Ltd., Durham University, Durham, UK), the structure was solved with the SIR2004 structure solution program using direct methods and refined with the XL refinement package using least squares minimization [23,24,25].

### 3.7. AchE Inhibitory Assay

The inhibitory activities against AchE of compounds **1**–**5** were investigated in vitro using the modified Ellman method [26]. The substrates were *S*-acetylthiocholine iodide and 5,5′-dithio-bis-(2-nitrobenzoic acid). Huperzine A was used as a positive control.

### 3.8. DPPH Radical Scavenging Assay

The radical scavenging effect on DPPH of compounds **1**–**5** were determined according to previously reported methods [27,28], and 2,6-ditertbutyl-4-methylphenol was used as a positive control.

### 3.9. OH-Radical-Scavenging Assay

Radical scavenging effect on OH of compounds **1**–**5** were carried out according to previously reported methods [29,30]. The indicator used was 1,10-phenanthroline-Fe^2+^; vitamin C was used as a positive control.

### 3.10. α-Glucosidase Inhibitory Assay

The inhibitory activities against α-glucosidase of compounds **1**–**5** were investigated in vitro using the modified method described by Moradi-Afrapoli et al. [31]; *p*-nitrophenyl-α-d-glucopyranoside was used as the substrates, and *trans*-resveratrol was used as a positive control.

### 3.11. Antibacterial Experiment

The antibacterial activity of compounds **1**–**5** were investigated in vitro using the modified 96 well microtiter-based method described by Pierce et al. [32].

## 4. Conclusions

Mangrove endophytic fungi from the South China Sea provide rich fungal diversity, and are promising sources of structurally-unprecedented bioactive natural products [33,34,35,36,37]. Five chromone derivatives were isolated from the mangrove endophytic fungus *Phomopsis* sp. 33#, four of them are new compounds (**1**–**4**). Compounds **1**–**5** showed weak inhibitory activity of AchE as well as α-glucosidase, radical scavenging effects on DPPH as well as OH, and low antimicrobial activity. The compounds (**1**–**4**) showed no cytotoxic activity against MDA-MB-435 breast cancer cells. Their other bioactivities are worthy of further study, considering their unique tricyclic molecular structures, in which a dihydropyran ring is fused with the chromone ring.

## Figures and Tables

**Table 1:** ^1^H-NMR and ^13^C-NMR data of compounds **1**–**4** (400/100 MHz, *J* in Hz).

	1 (in C_3_D_6_O)		2 (in CDCl_3_)		3 (in CDCl_3_)		4 (in CD_3_OD)	
	*δ*_C_	*δ*_H_	*δ*_C_	*δ*_H_	*δ*_C_	*δ*_H_	*δ*_C_	*δ*_H_
1	21.0 q	1.34 d 6.0	21.0 q	1.39 d 6.4	21.3 q	1.38 d 6.0	23.8 q	1.31 d 6.0
2	62.8 d	4.34 m	62.1 d	4.41 m	70.0 d	3.83 m	66.7 t	4.23 m
3	34.6 t	2.67 dd 18.0, 4.0	34.4 t	2.63 m	34.6 t	2.64 m	42.1 t	2.94 m
		2.58 dd 18.0,10.8						
4	164.1 s		163.6 s		160.7 s		167.7 s	
5	158.4 s		144.4 s		144.8 s		159.3 s	
6	104.3 d	6.93 d 2.4	134.7 s		134.9 s		104.4 d	6.87 d 2.4
7	162.6 s		149.2 s		149.0 s		164.2 s	
8	113.8 d	6.85 d 2.4	108.0 d	6.89 s	107.9 d	6.93 s	114.8 d	6.77 d 2.4
9	114.6 s		124.5 s		124.0 s		113.7 s	
10	136.4 s		116.0 s		115.6 s		136.2 s	
11	173.2 s		173.5 s		174.1 s		177.2 s	
12	117.4 s		116.6 s		116.5 s		122.1 s	
13	95.2 d	5.40 s	94.5 d	5.57 s	62.5 t	4.82 d 15.2	55.0 t	4.55 s
						4.48 d 15.2		
14	169.5 s		169.8 s		170.0 s		171.6 s	
15	52.8 q	3.85 s	53.2 q	3.95 s	53.2 q	3.96 s	53.3 q	3.91 s
16	55.6 q	3.42 s	55.9 q	3.55 s				
17			56.8 q	3.98 s	56.9 q	3.99 s		

## Supplementary Materials

The following are available online at www.mdpi.com/1660-3397/14/11/215/s1. Figure S1: ^1^H-NMR for phomopsichin A (**1**). Figure S2: ^13^C-NMR for phomopsichin A (**1**). Figure S3: ^1^H-^1^H COSY for phomopsichin A (**1**). Figure S4: heteronuclear single quantum coherence (HSQC) spectroscopy for phomopsichin A (**1**). Figure S5: HMBC for phomopsichin A (**1**). Figure S6: NOESY for phomopsichin A (**1**). Figure S7: HR mass spectrometry for phomopsichin A (**1**). Figure S8: ^1^H-NMR for phomopsichin B (**2**). Figure S9: ^13^C-NMR for phomopsichin B (**2**). Figure S10: ^1^H-^1^H COSY for phomopsichin B (**2**). Figure S11: HSQC for phomopsichin B (**2**). Figure S12: HMBC for phomopsichin B (**2**). Figure S13: NOESY for phomopsichin B (**2**). Figure S14: HR mass spectrometry for phomopsichin B (**2**). Figure S15: ^1^H-NMR for phomopsichin C (**3**). Figure S16: ^13^C-NMR for phomopsichin C (**3**). Figure S17: ^1^H-^1^H COSY for phomopsichin C (**3**). Figure S18: HSQC for phomopsichin C (**3**). Figure S19: HMBC for phomopsichin C (**3**). Figure S20: HR mass spectrometry for phomopsichin C (**3**). Figure S21: ^1^H-NMR for phomopsichin D (**4**). Figure S22: ^13^C-NMR for phomopsichin D (**4**). Figure S23: ^1^H-^1^H COSY for phomopsichin D (**4**). Figure S24: HSQC for phomopsichin D (**4**). Figure S25: HMBC for phomopsichin D (**4**). Figure S26: HR mass spectrometry for phomopsichin D (**4**). Figure S27: ECD spectrometry for phomoxanthone A (**5**). Table S1: Crystal data and structure refinement for phomopsichin A (**1**). Table S2: Fractional atomic coordinates (× 10^4^) and equivalent isotropic displacement parameters (Å^2^ × 10^3^) for phomopsichin A (**1**). Table S3: Anisotropic displacement parameters (Å^2^ × 10^3^) for phomopsichin A (**1**). Table S4: Bond lengths for phomopsichin A (**1**). Table S5: Bond angles for phomopsichin A (**1**). Table S6: Torsion angles for phomopsichin A (**1**). Table S7: Hydrogen atom coordinates (Å × 10^4^) and isotropic displacement parameters (Å^2^ × 10^3^) for phomopsichin A (1). Table S8: Inhibitory activities against AchE as well as α-glucosidase, and the radical scavenging effects on DPPH as well as OH of compounds **1**–**5**. Table S9: Antimicrobial activity of compounds **1**–**5**.
