# Supplementary material for: Phomopsichin A–D; Four New Chromone Derivatives from Mangrove Endophytic Fungus Phomopsis sp. 33#
Source: Mar Drugs. 2016 Nov 22;14(11):215. doi: 10.3390/md14110215 (PMC5128758; doi:10.3390/md14110215)
Supplement: Supplementary file 1 [file marinedrugs-14-00215-s001.pdf]

Supplementary Materials: Phomopsichin A–D; Four New Chromone Derivatives from Mangrove Endophytic Fungus *Phomopsis* sp. 33#

Meixiang Huang, Jing Li, Lan Liu, Sheng Yin, Jun Wang and Yongcheng Lin

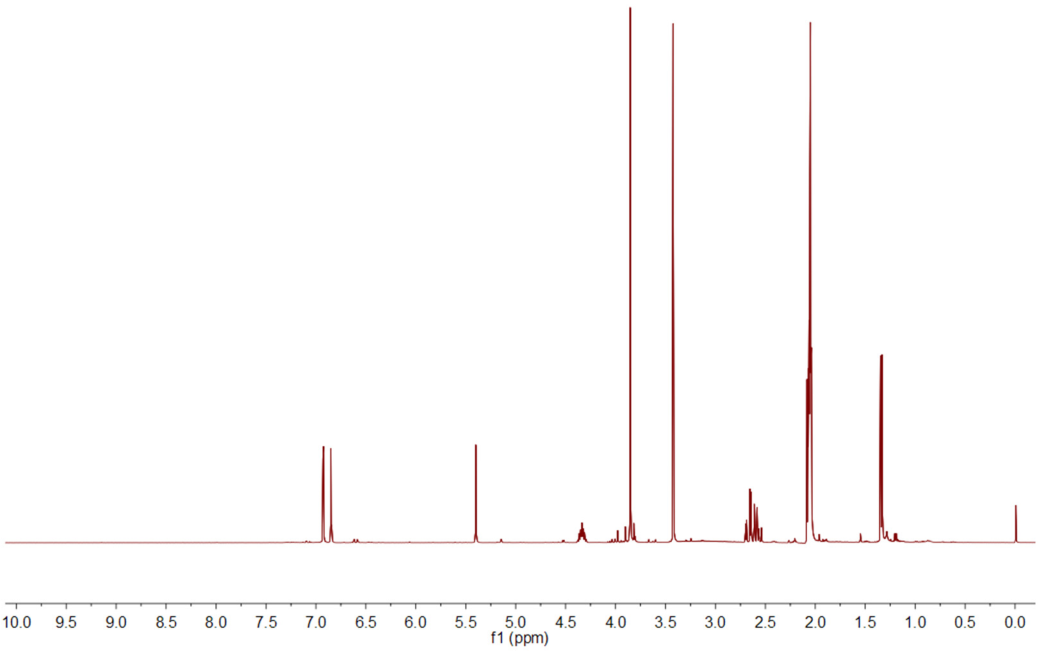

Figure S1. ^1H NMR for phomopsichin A (**1**).

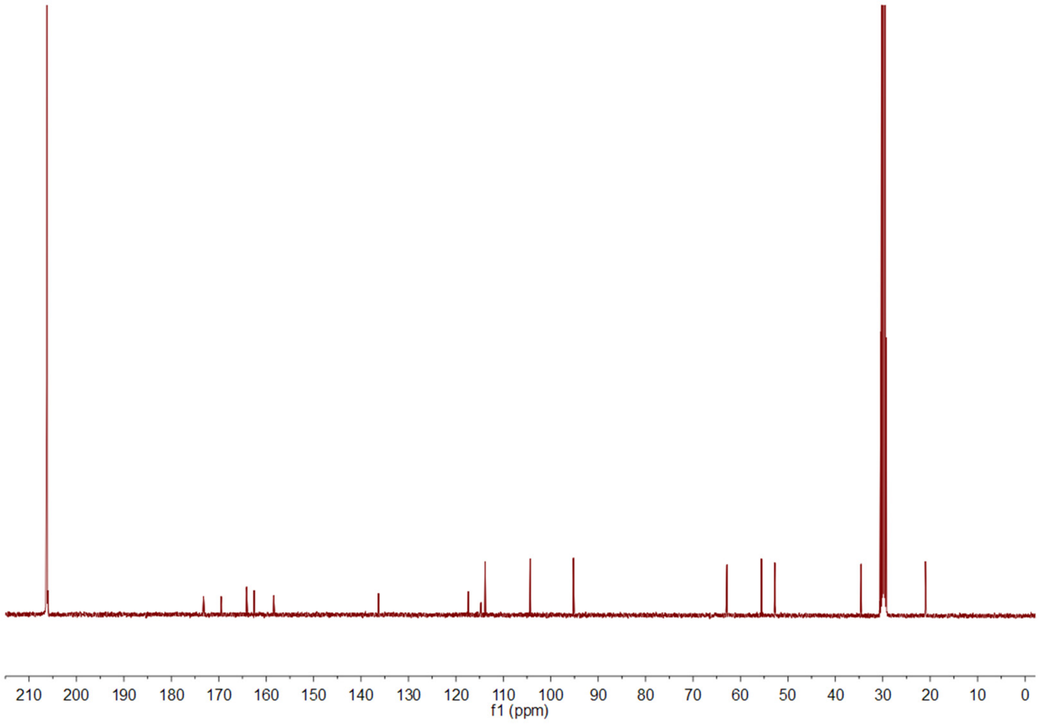

Figure S2. ^{13}C NMR for phomopsichin A (**1**).

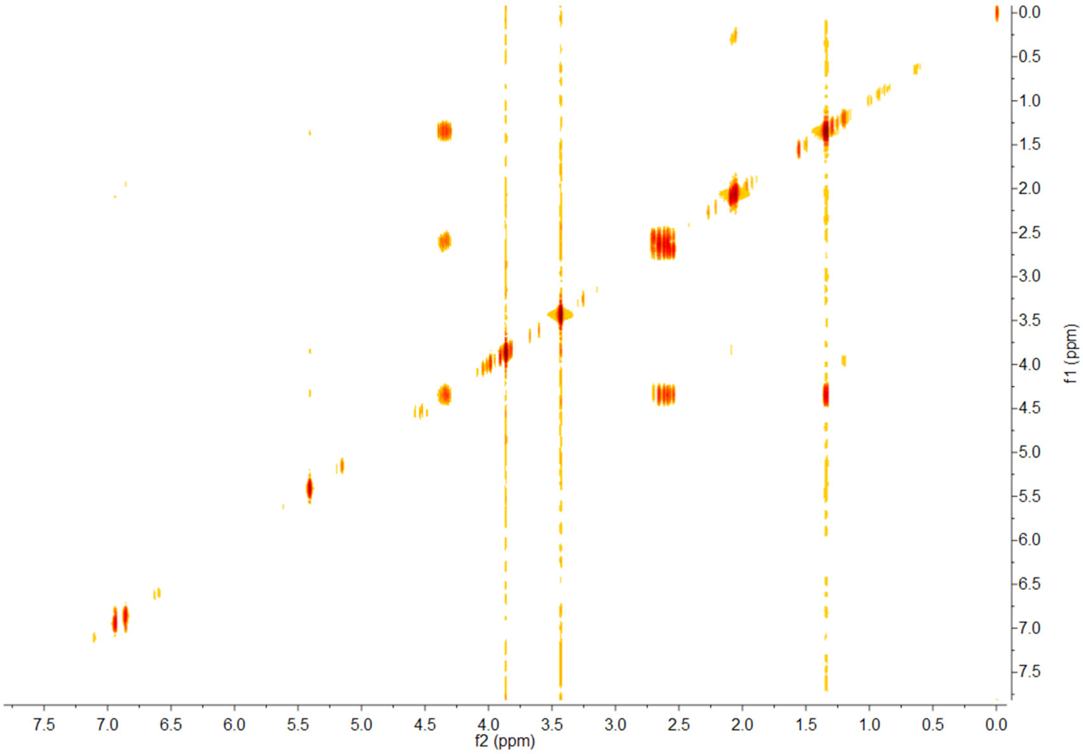

Figure S3. ^{1}H-^{1}H COSY for phomopsichin A (**1**).

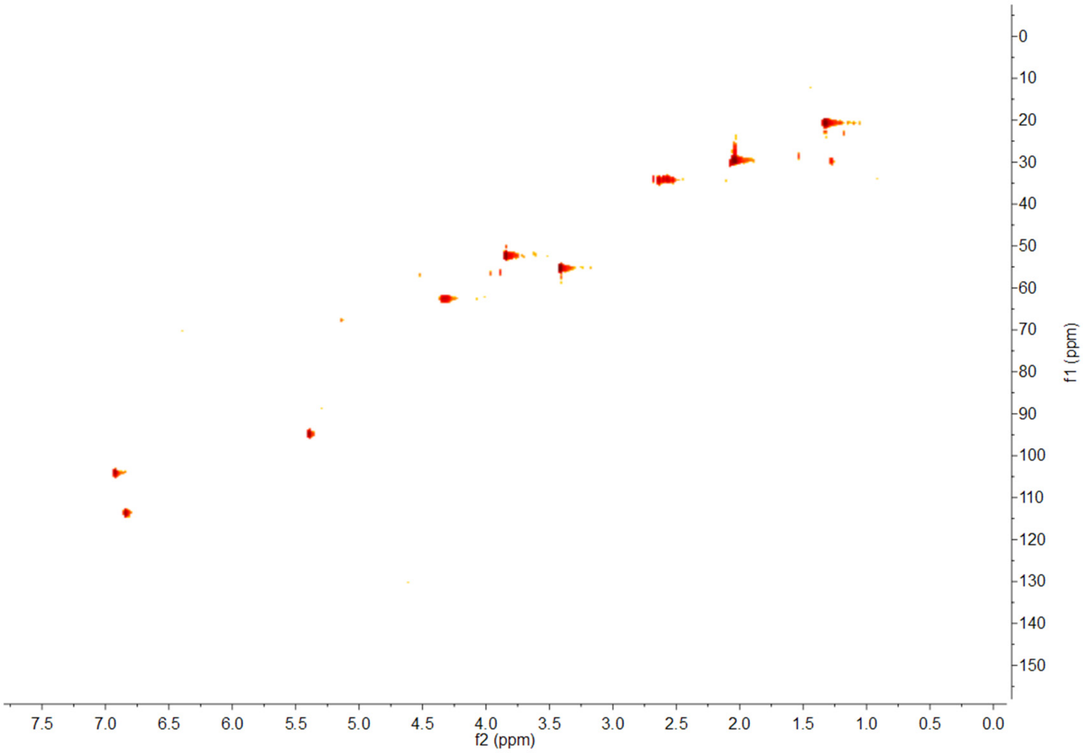

Figure S4. HSQC for phomopsichin A (**1**).

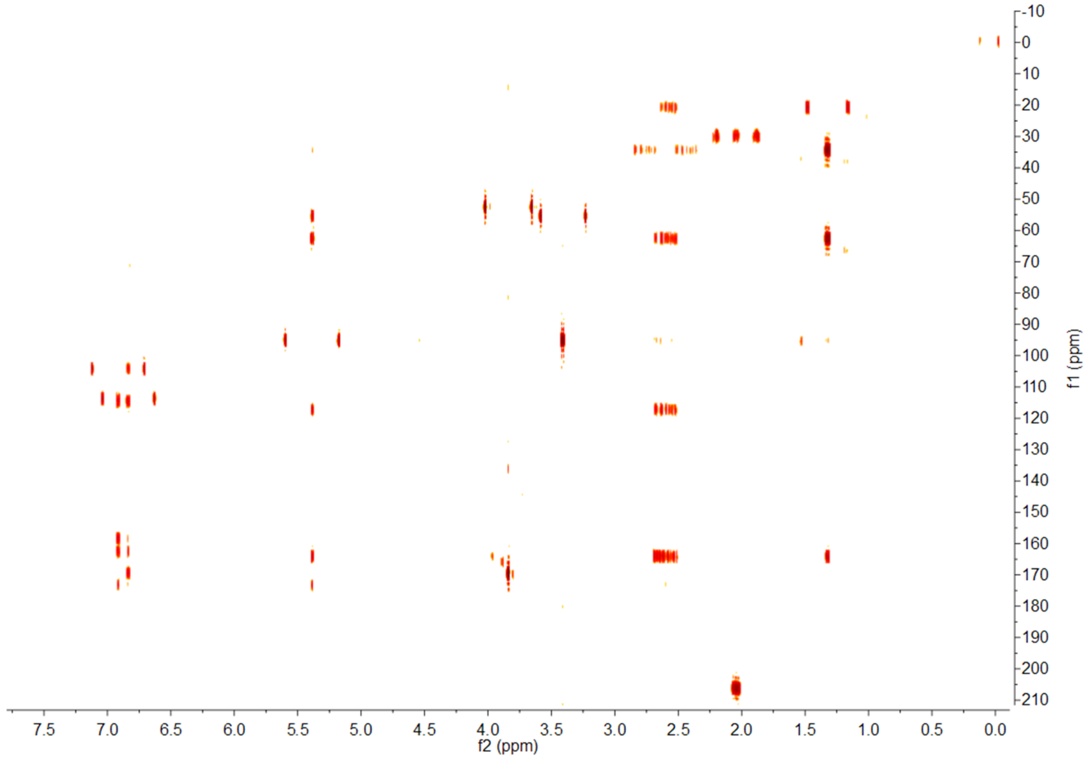

Figure S5. HMBC for phomopsichin A (**1**).

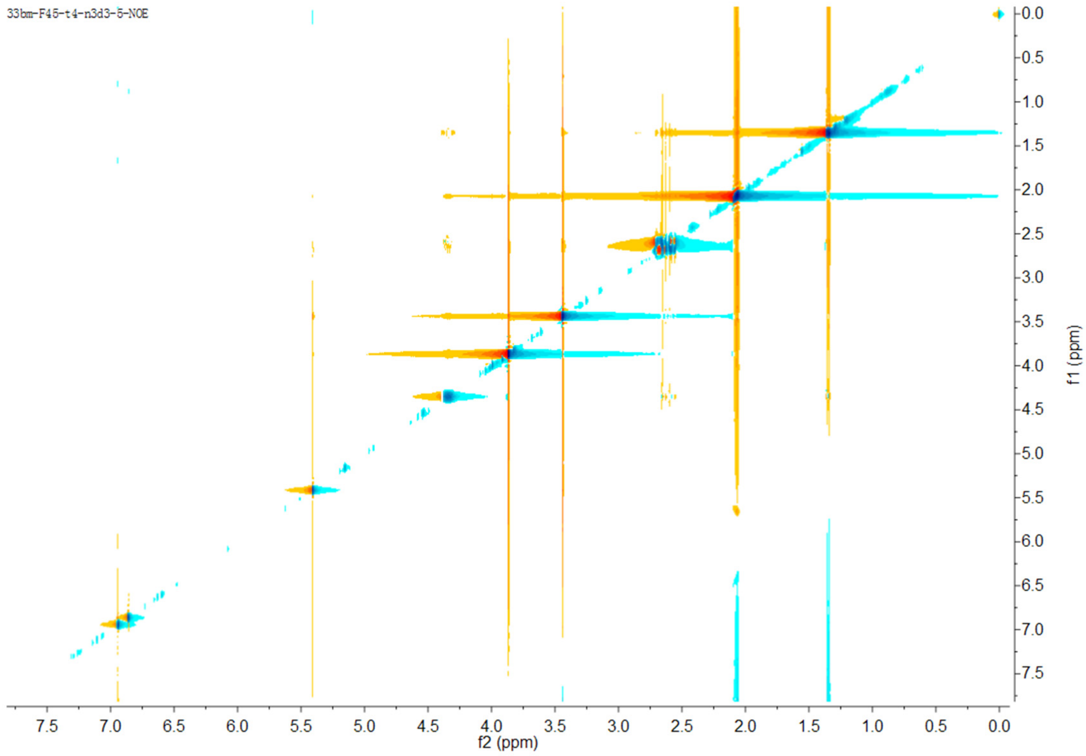

Figure S6. NOESY for phomopsichin A (**1**).

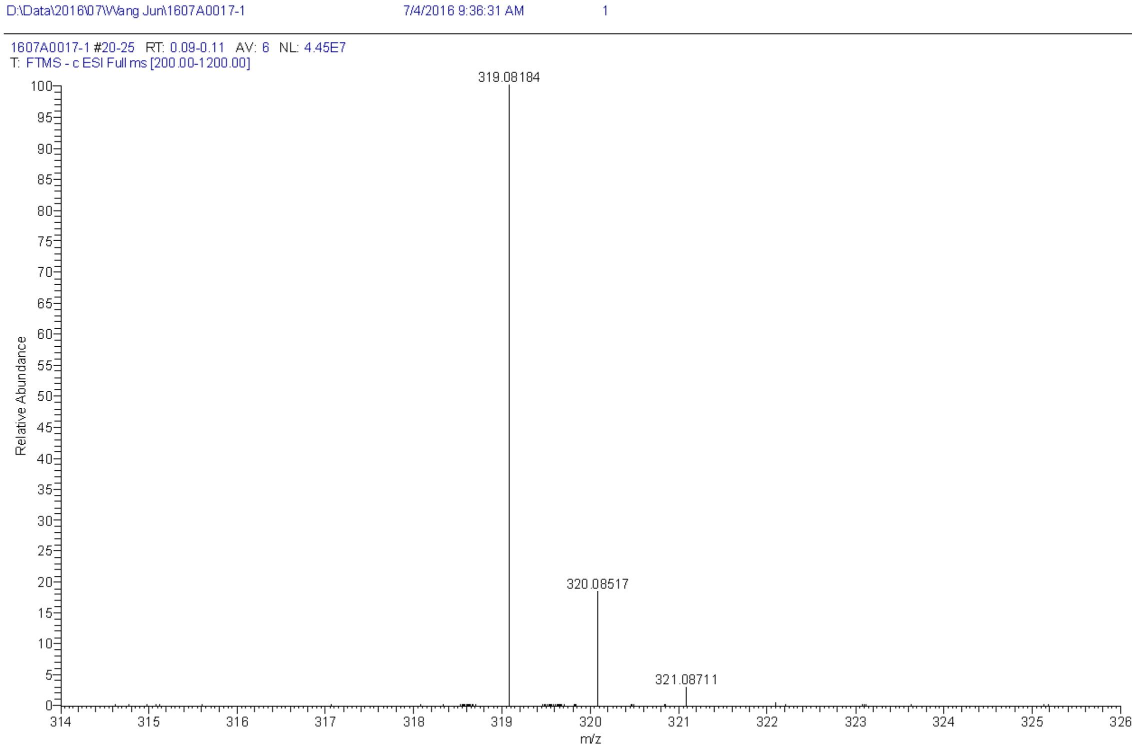

SPECTRUM - simulation :

m/z	Theo. Mass	Delta (ppm)	RDB equiv.	Composition
319.08184	319.08233	-1.52	9.5	$C_{16}H_{15}O_7$

Limits:

(1) Charge: -1
(2) Nitrogen-Rule: Do not use
(3) Mass tolerance: 10.00 ppm

Elements in use: ^{12}C (0~20), ^{1}H (0~30), ^{16}O (0~10), ^{14}N (0~3)

Figure S7. HR mass spectrometry for phomopsichin A (**1**).

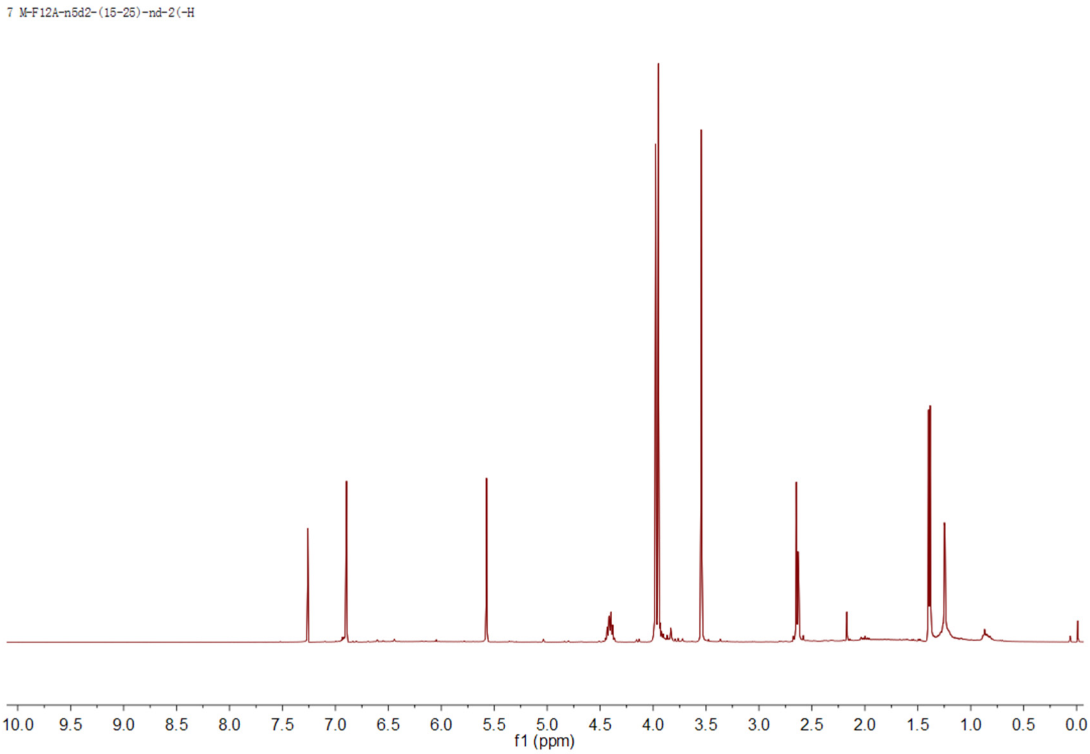

Figure S8. ^{1}H NMR for phomopsichin B (**2**).

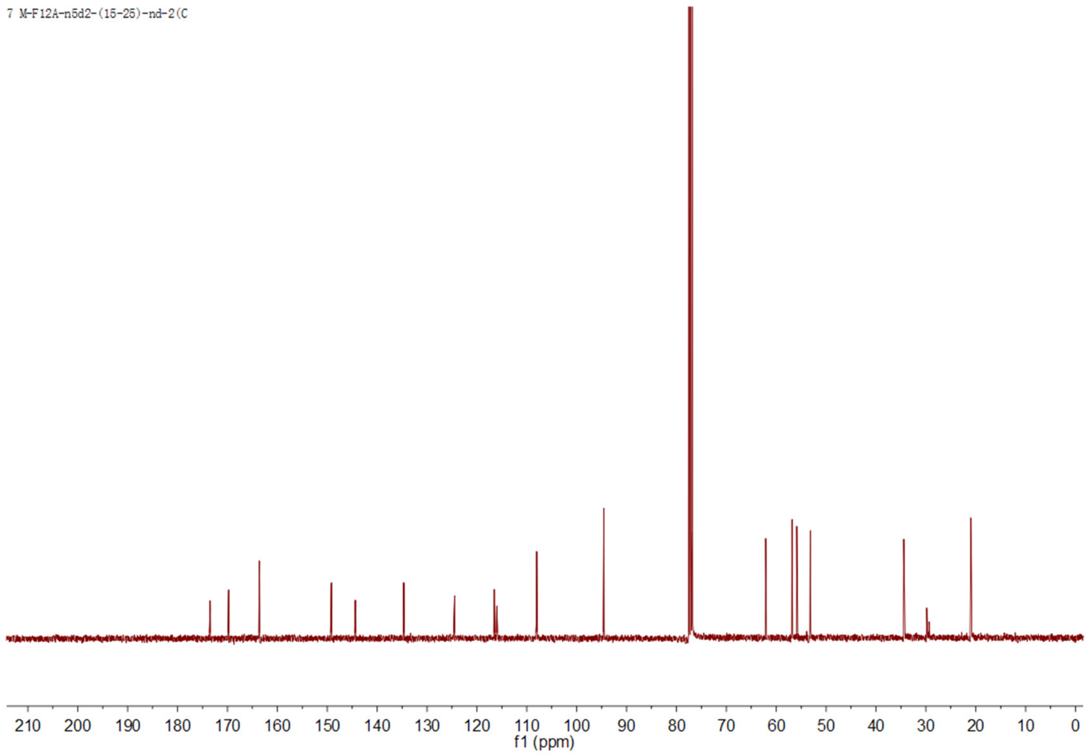

Figure S9. ^{13}C NMR for phomopsichin B (**2**).

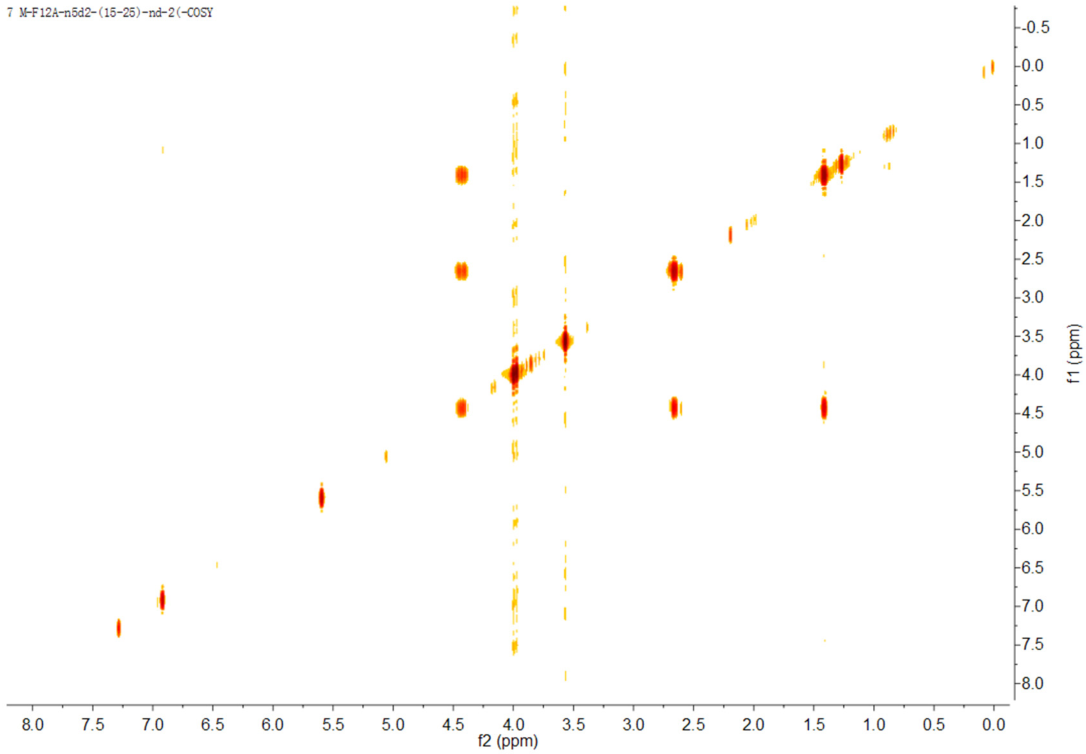

Figure S10. ^{1}H-^{1}H COSY for phomopsichin B (**2**).

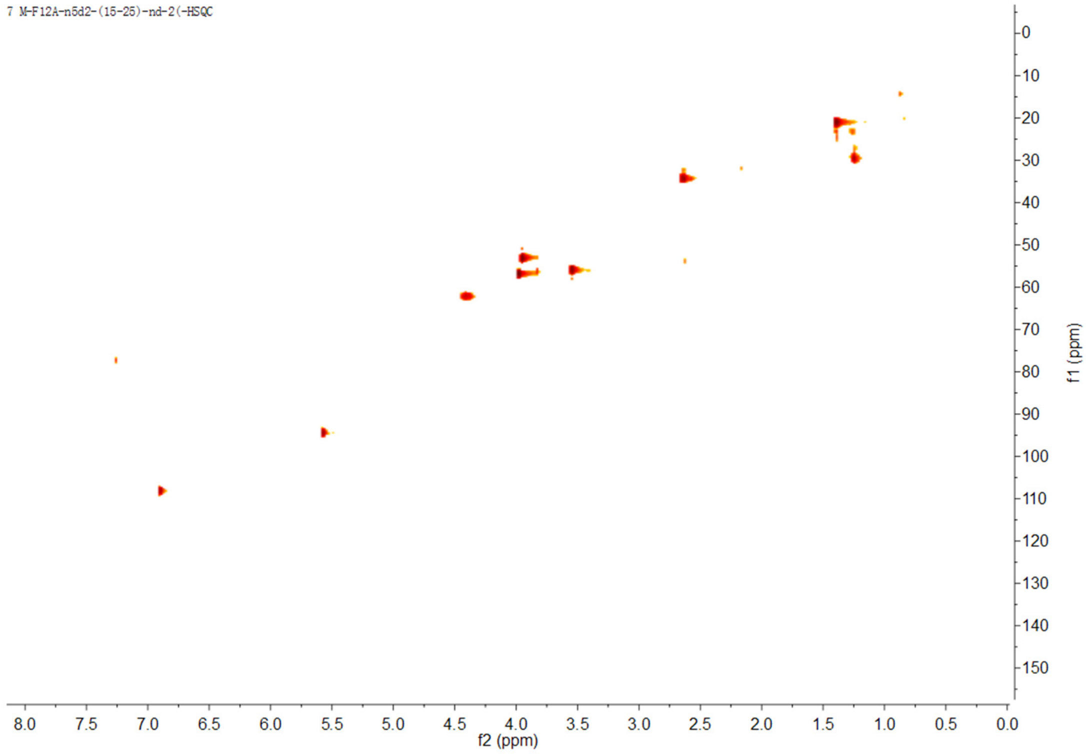

Figure S11. HSQC for phomopsichin B (**2**).

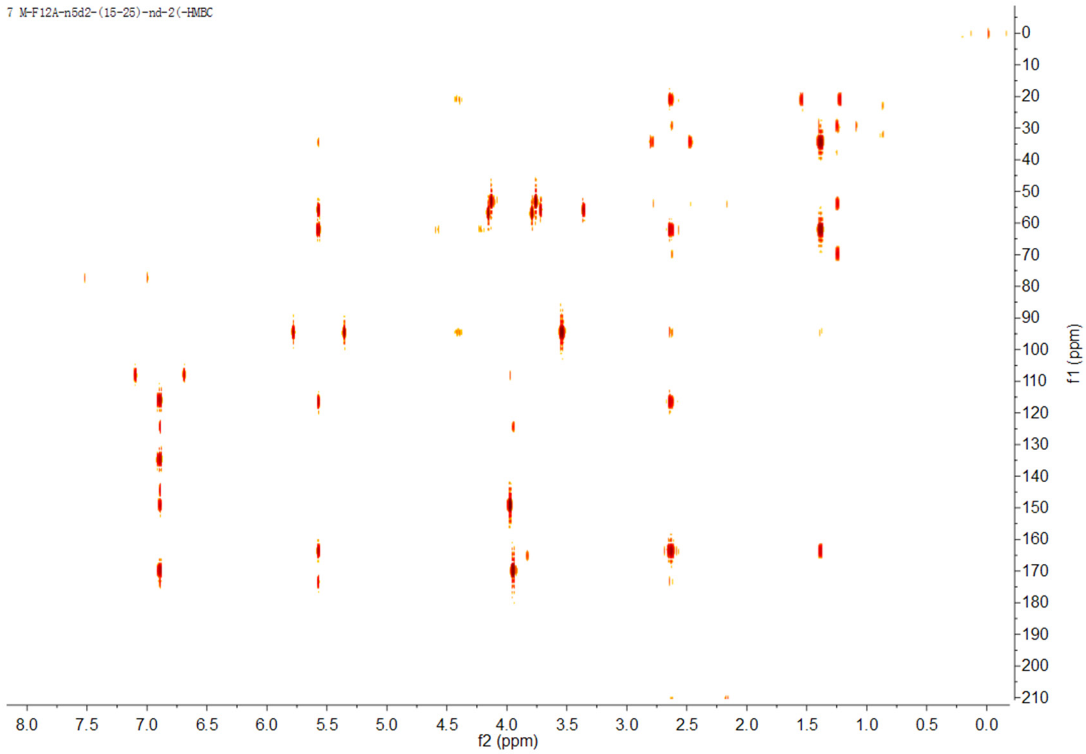

Figure S12. HMBC for phomopsichin B (**2**).

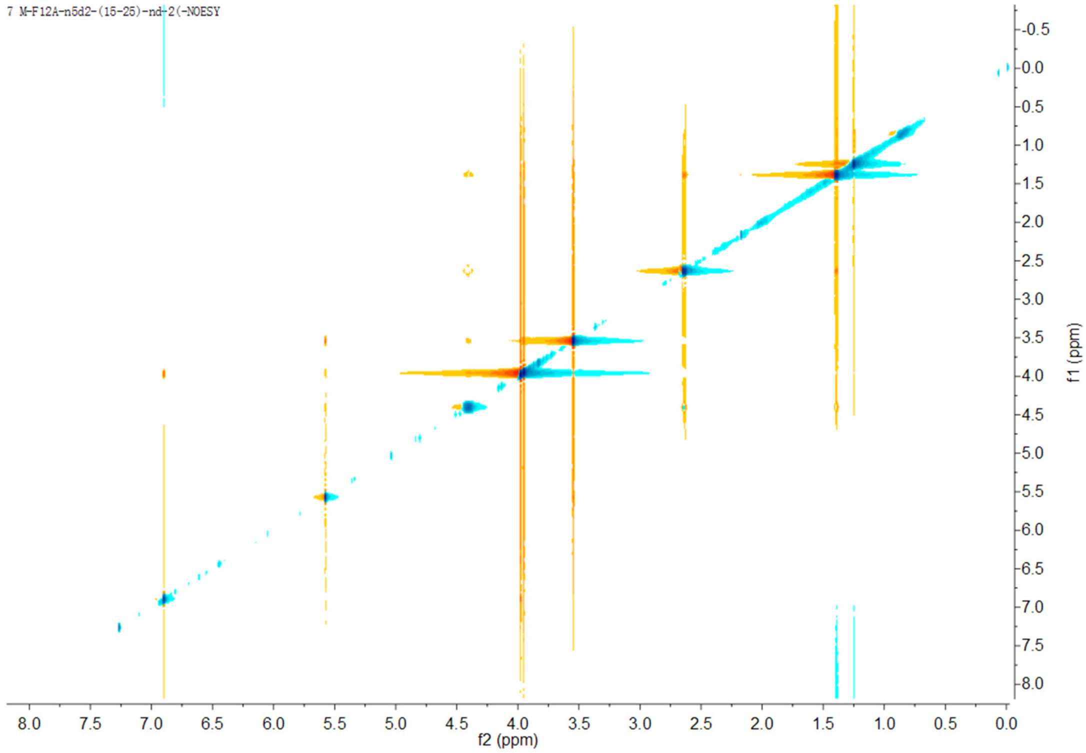

Figure S13. NOESY for phomopsichin B (**2**).

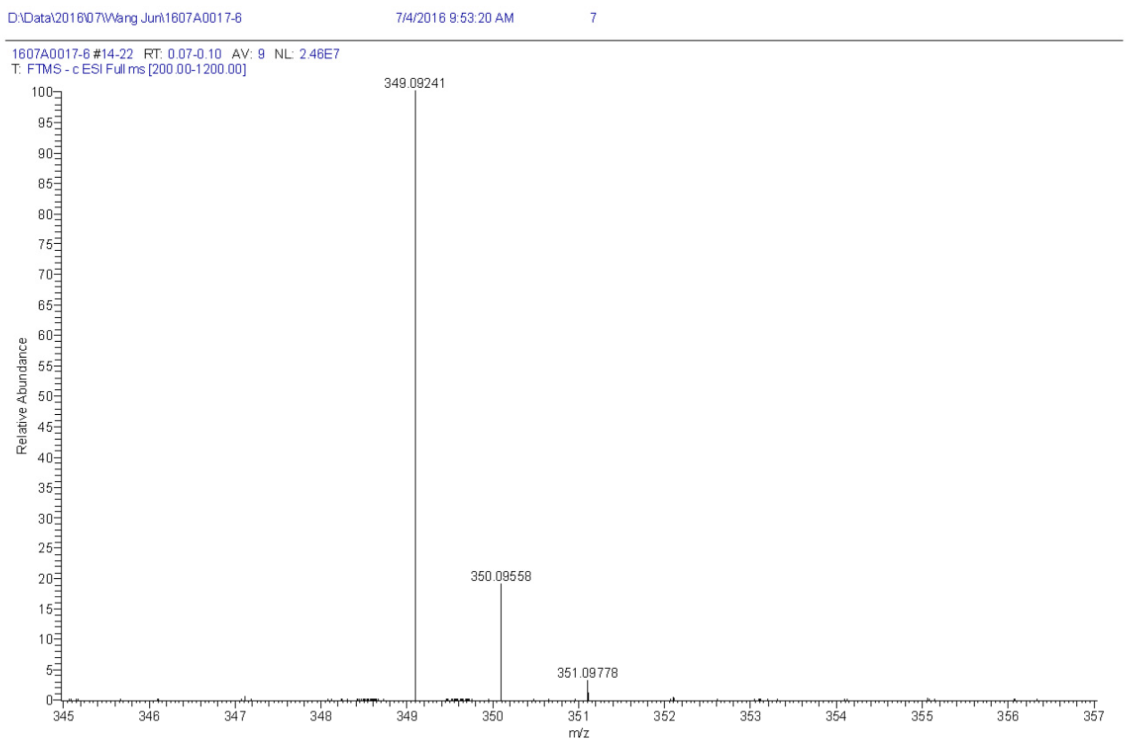

SPECTRUM - simulation :

m/z	Theo. Mass	Delta (ppm)	RDB equiv.	Composition
349.09241	349.09289	-1.38	9.5	$C_{17}H_{17}O_8$

Limits:

(1) Charge: -1
(2) Nitrogen-Rule: Do not use
(3) Mass tolerance: 10.00 ppm

Elements in use: ^{12}C (0~20), ^{1}H (0~30), ^{16}O (0~10), ^{14}N (0~3)

Figure S14. HR mass spectrometry for phomopsichin B (**2**).

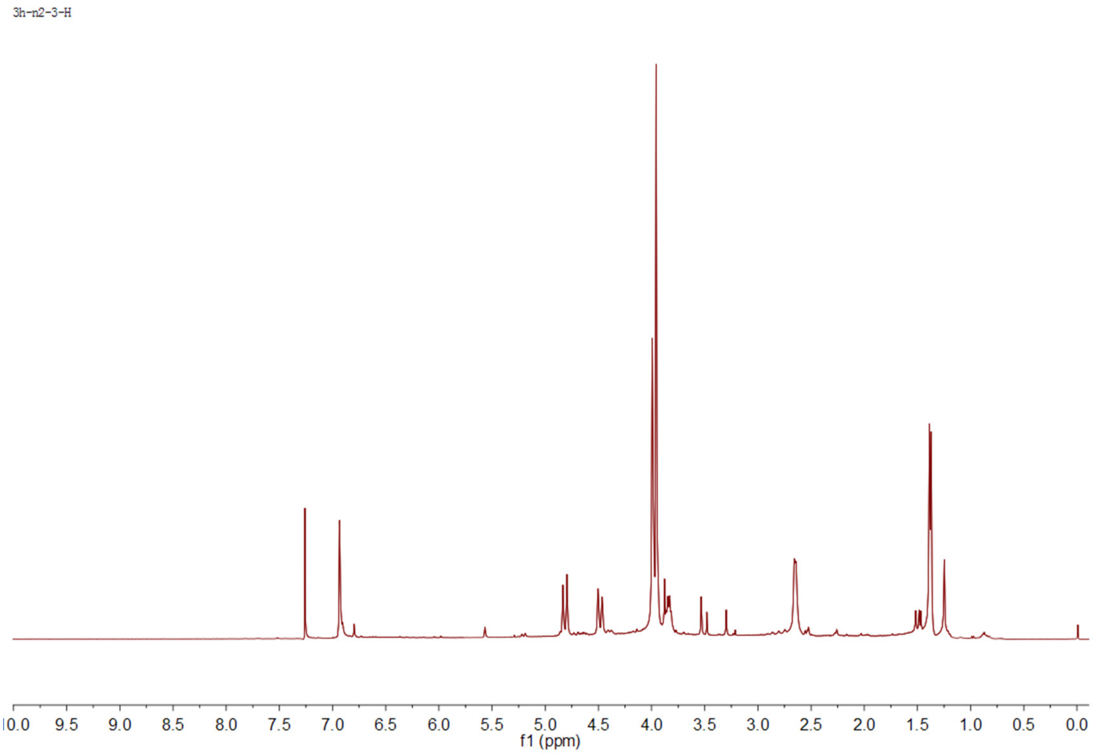

Figure S15. ^{1}H NMR for phomopsichin C (**3**).

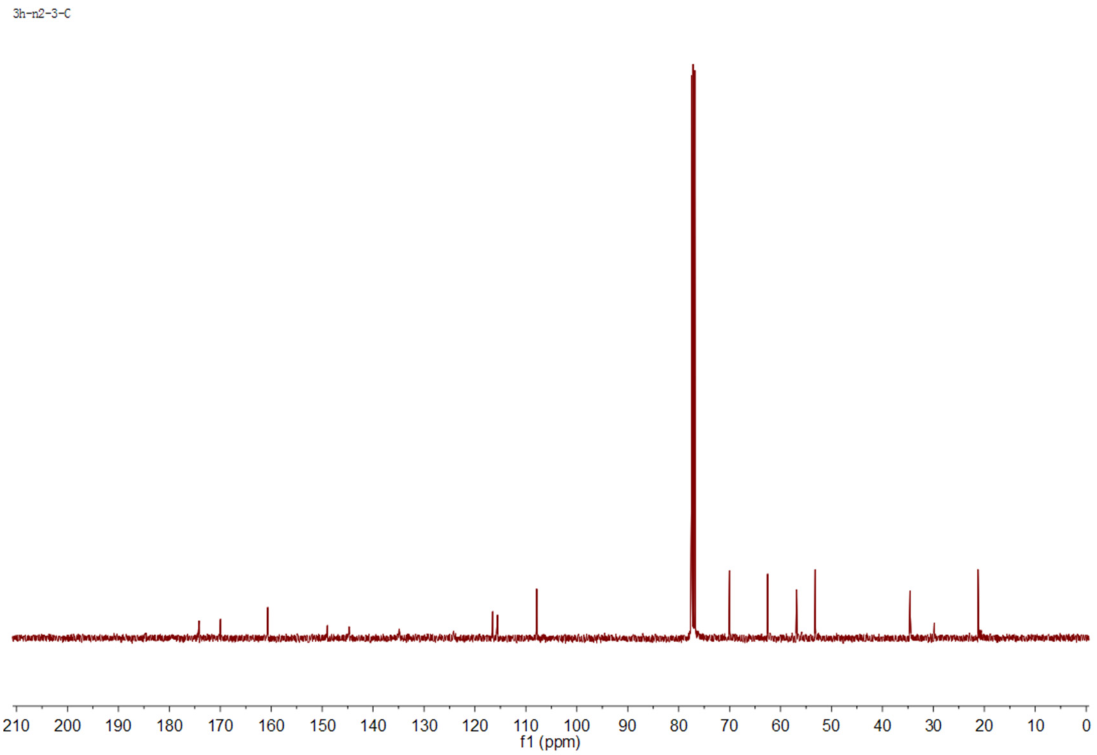

Figure S16. ^{13}C NMR for phomopsichin C (**3**).

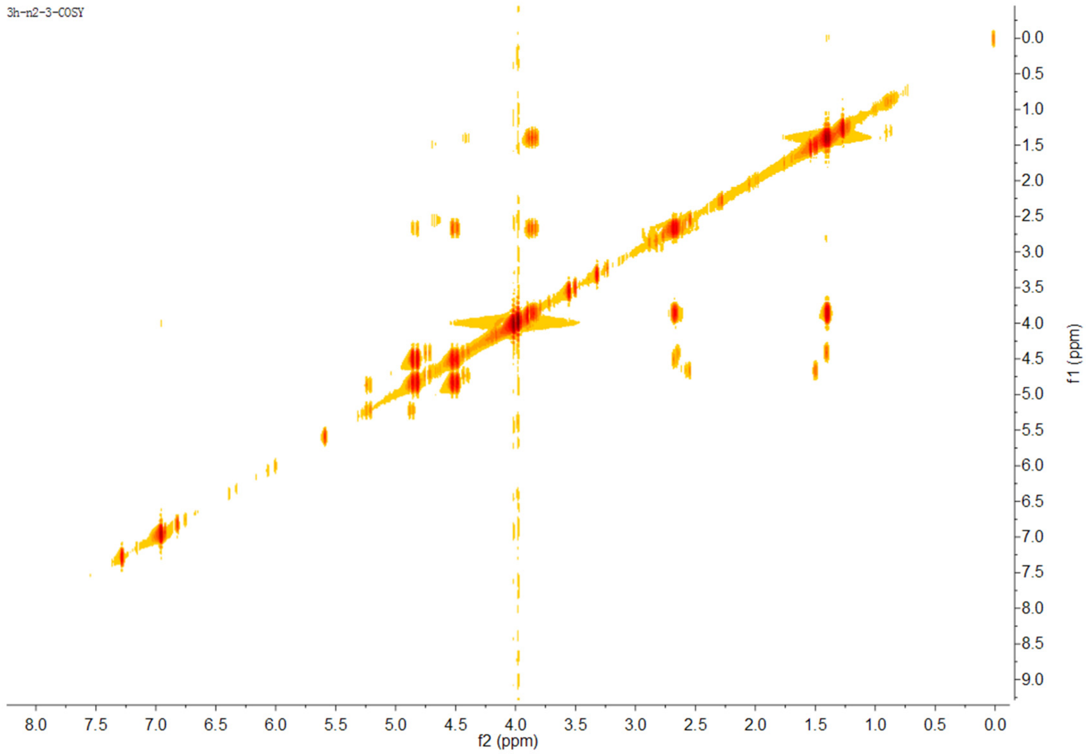

Figure S17. ^{1}H-^{1}H COSY for phomopsichin C (**3**).

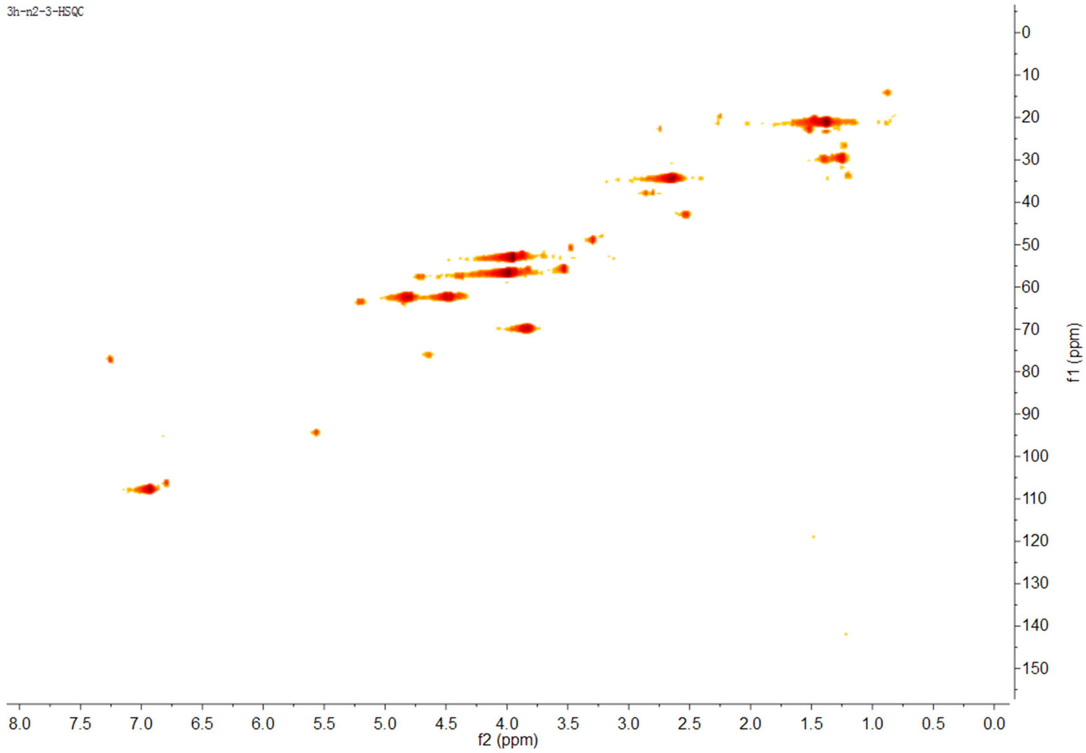

Figure S18. HSQC for phomopsichin C (**3**).

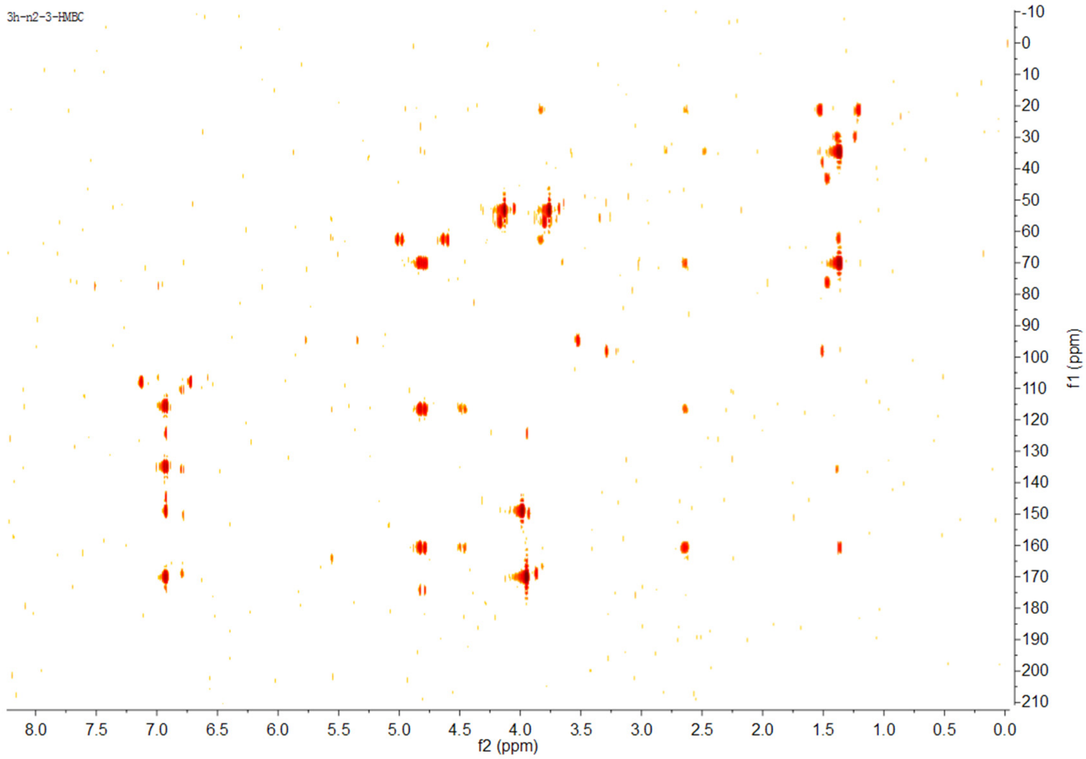

Figure S19. HMBC for phomopsichin C (**3**).

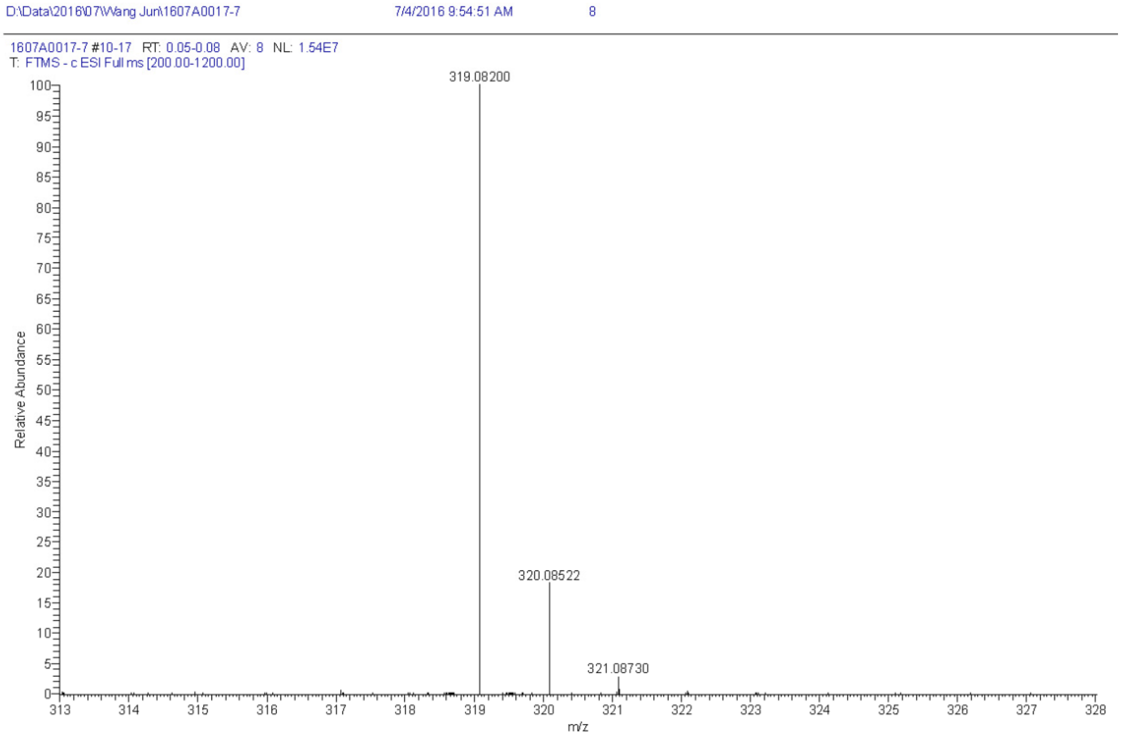

SPECTRUM - simulation :

m/z	Theo. Mass	Delta (ppm)	RDB equiv.	Composition
319.08200	319.08233	-1.02	9.5	$C_{16}H_{15}O_7$

Limits:

(1) Charge: -1
(2) Nitrogen-Rule: Do not use
(3) Mass tolerance: 10.00 ppm

Elements in use: ^{12}C (0~20), ^{1}H (0~30), ^{16}O (0~10), ^{14}N (0~3)

Figure S20. HR mass spectrometry for phomopsichin C (**3**).

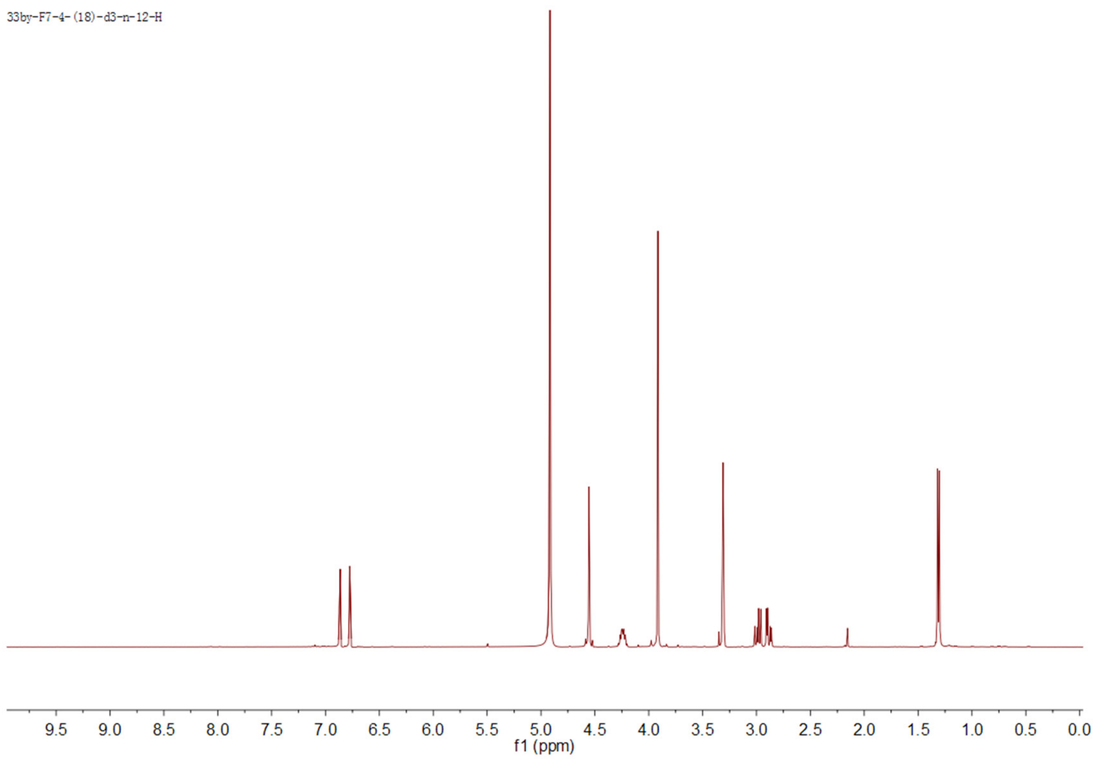

Figure S21. ^{1}H NMR for phomopsichin D (**4**).

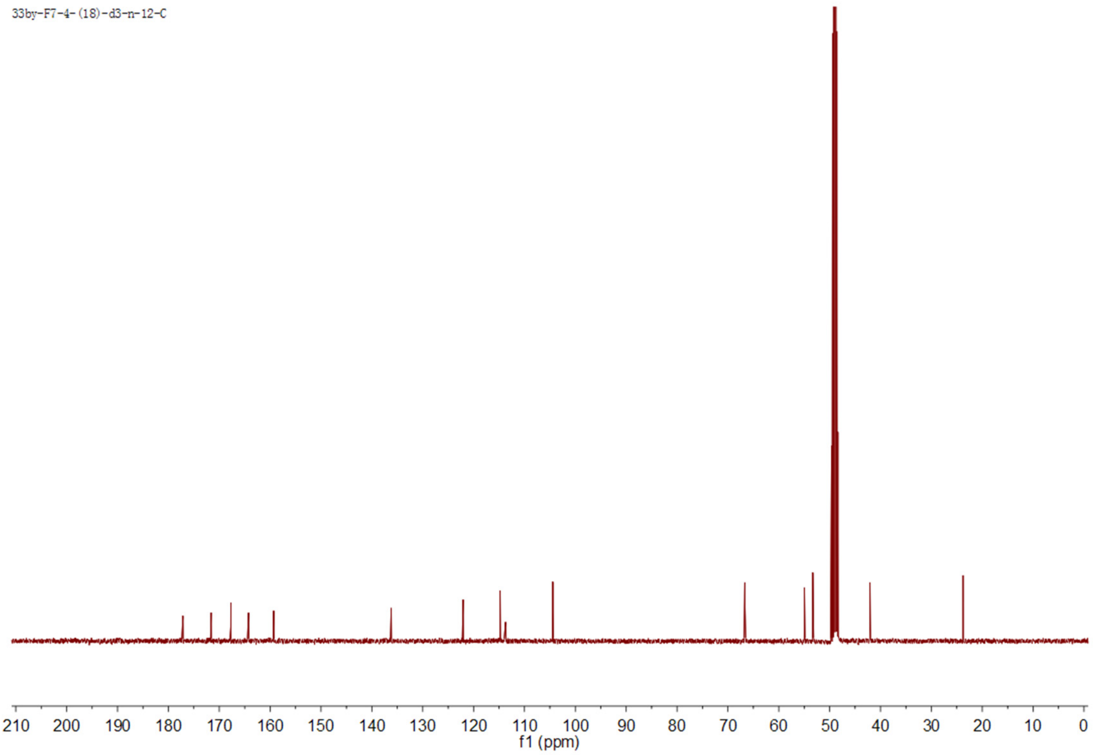

Figure S22. ^{13}C NMR for phomopsichin D (**4**).

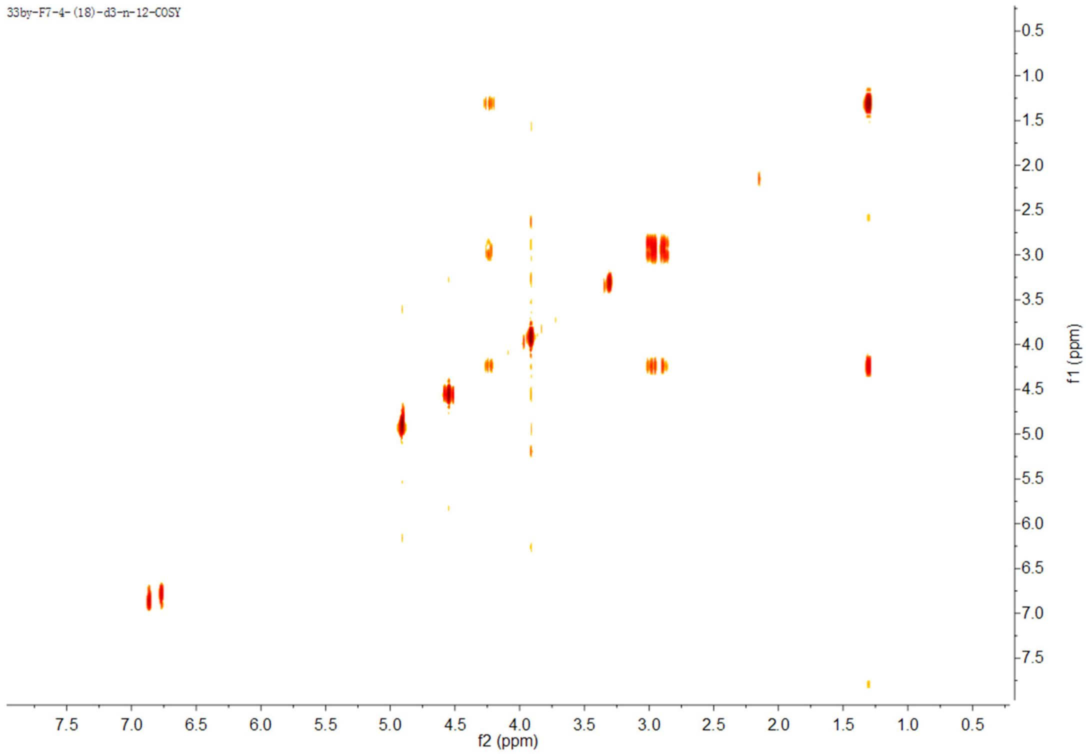

Figure S23. ^{1}H-^{1}H COSY for phomopsichin D (**4**).

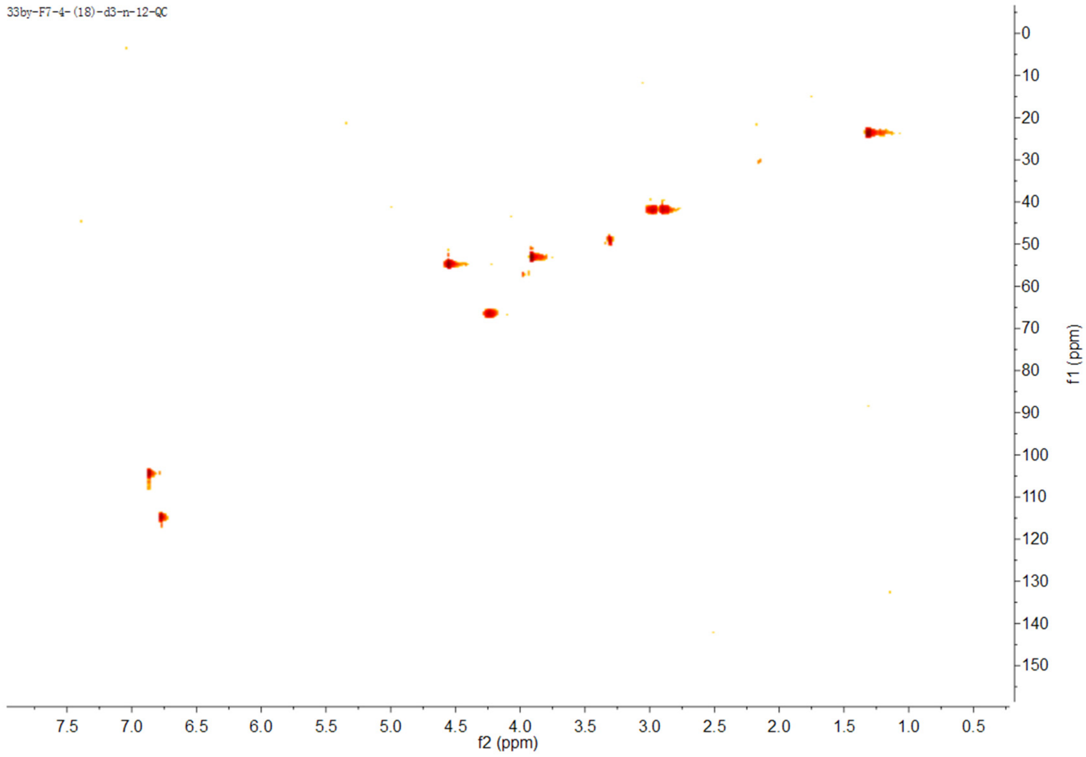

Figure S24. HSQC for phomopsichin D (**4**).

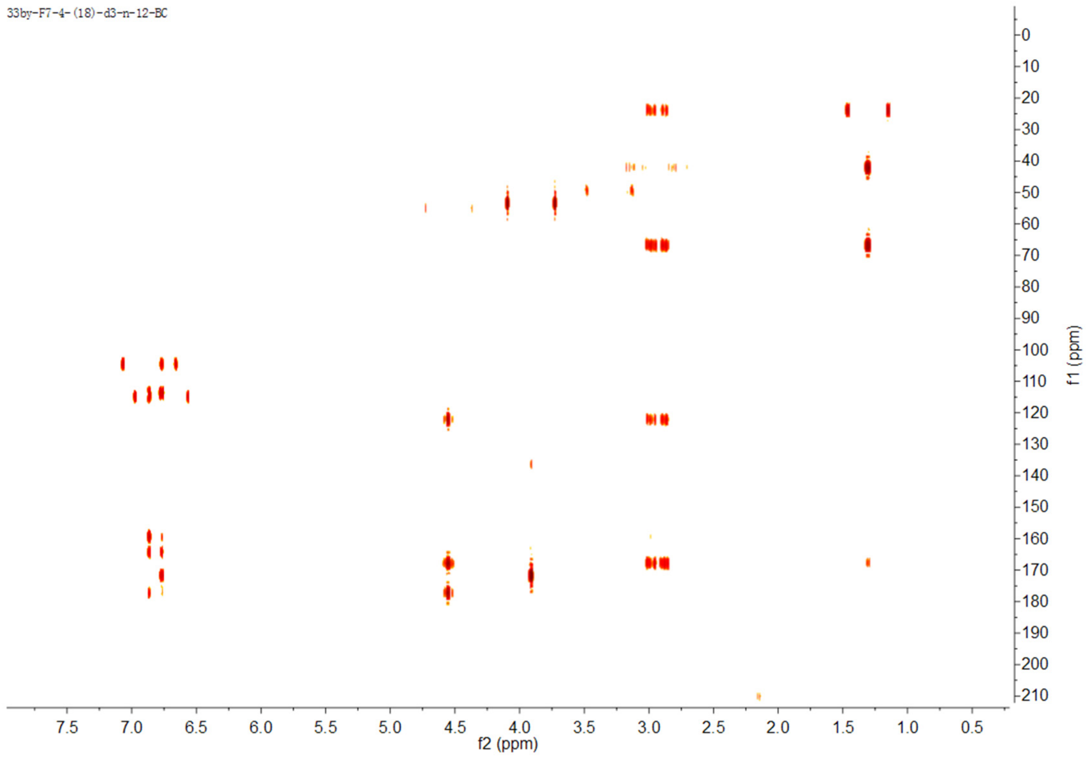

Figure S25. HMBC for phomopsichin D (**4**).

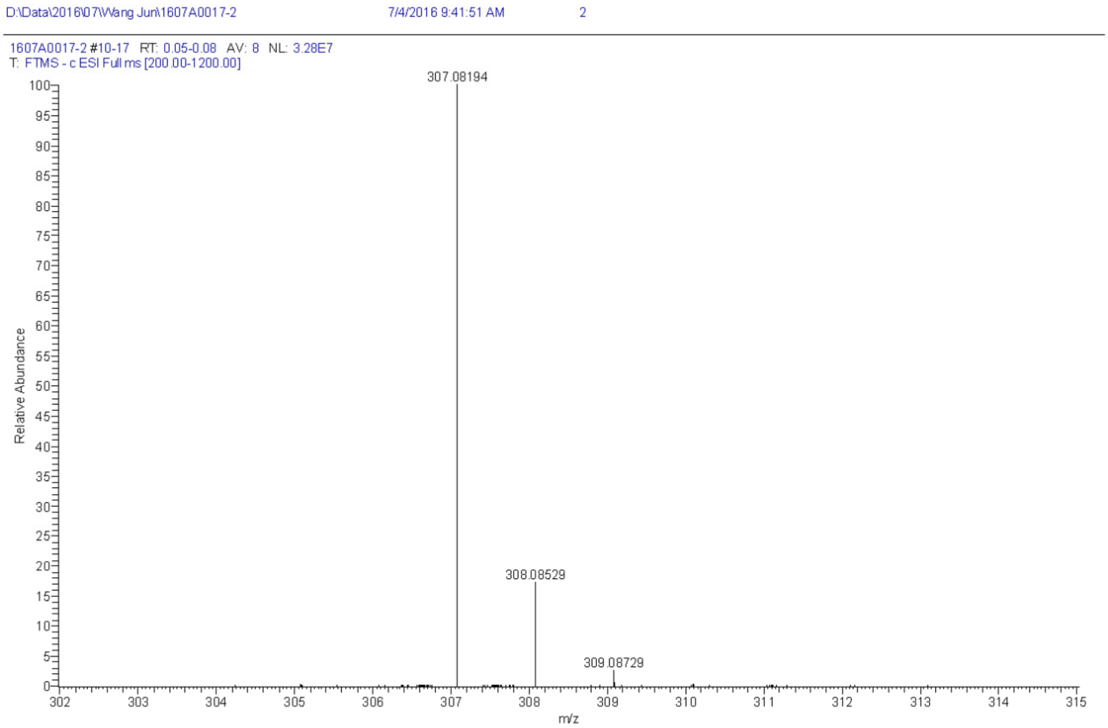

SPECTRUM - simulation :

m/z	Theo. Mass	Delta (ppm)	RDB equiv.	Composition
307.08194	307.08233	-1.26	8.5	$C_{15}H_{15}O_7$

Limits:

(1) Charge: -1
(2) Nitrogen-Rule: Do not use
(3) Mass tolerance: 10.00 ppm

Elements in use: ^{12}C (0~20), ^{1}H (0~30), ^{16}O (0~10), ^{14}N (0~3)

Figure S26. HR mass spectrometry for phomopsichin D (**4**).

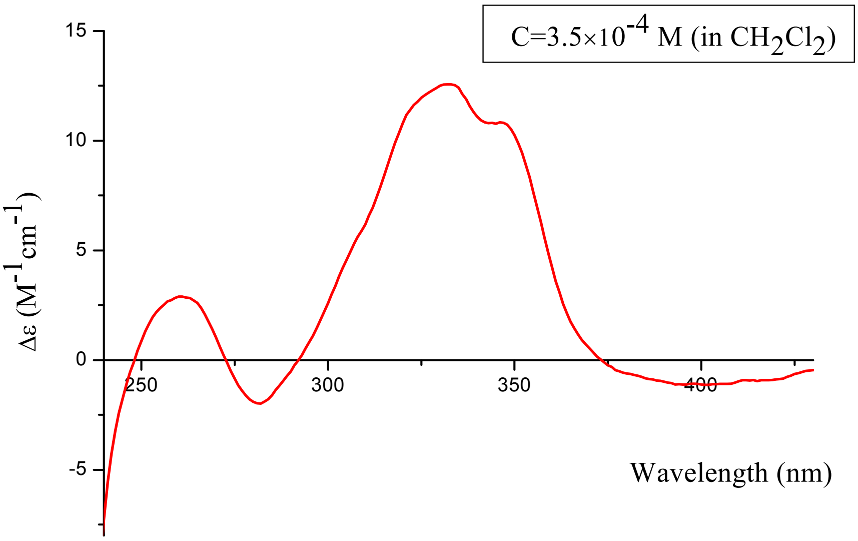

Figure S27. ECD spectra for phomoxanthone A (**5**).

Table S1. Crystal data and structure refinement for phomopsichin A (**1**).

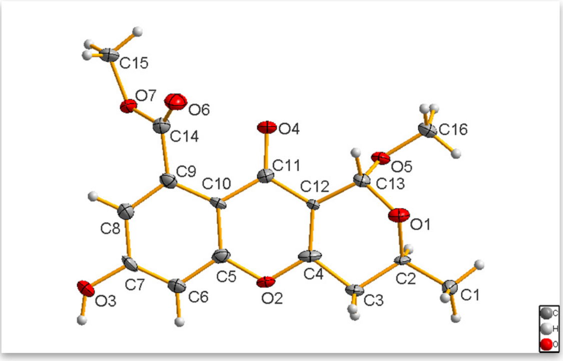

Identification code	exp_9763
Empirical formula	$C_{16}H_{16}O_7$
Formula weight	320.29
Temperature/K	150(2)
Crystal system	monoclinic
Space group	$P2_1$
a/Å	7.6234(9)
b/Å	15.5426(17)
c/Å	12.4889(13)
α/°	90.00
β/°	96.241(10)
γ/°	90.00
Volume/Å^3	1471.0(3)
Z	4
ρ_{calc}g/cm^3	1.446
μ/mm^{-1}	0.973
F(000)	672.0
Crystal size/mm^3	0.40 × 0.35 × 0.30
Radiation	CuKα (λ = 1.54178)
2Θ range for data collection/°	7.12 to 133.92
Index ranges	−8 ≤ h ≤ 6, −18 ≤ k ≤ 18, −14 ≤ l ≤ 14
Reflections collected	15309
Independent reflections	5018 [R_{int} = 0.0877, R_{sigma} = N/A]
Data/restraints/parameters	5018/0/421
Goodness-of-fit on F^2	1.109
Final R indexes [I>=2σ (I)]	R_1 = 0.0849, wR_2 = 0.2154
Final R indexes [all data]	R_1 = 0.0959, wR_2 = 0.2334
Largest diff. peak/hole/e Å^{-3}	0.54/−0.48
Flack parameter	0.02(3)

Table S2. Fractional atomic coordinates (× 10^4) and equivalent isotropic displacement parameters (Å^2 × 10^3) for phomopsichin A (**1**). U_{eq} is defined as 1/3 of of the trace of the orthogonalised U_{IJ} tensor.

–	*x*	*y*	*z*	**U(eq)**
C1	5200(14)	4098(6)	−508(7)	40(2)
C2	4749(13)	4333(6)	608(7)	33.1(19)
C3	4818(13)	3615(6)	1390(7)	37(2)
C4	4834(11)	3961(6)	2503(8)	37(2)
C5	4842(12)	3540(6)	4296(7)	33.5(19)
C6	4729(13)	2847(5)	5010(6)	40(2)

C7	4936(14)	2983(6)	6094(8)	44(2)
C8	5240(15)	3805(2)	6496(8)	44(2)
C9	5343(12)	4504(6)	5819(8)	34(2)
C10	5145(12)	4380(5)	4685(7)	30.4(18)
C11	5138(13)	5051(6)	3887(7)	38(2)
C12	5107(13)	4807(6)	2754(6)	31.8(19)
C13	5462(12)	5426(6)	1922(7)	33.9(19)
C14	5870(13)	5355(6)	6302(7)	39(2)
C15	5310(20)	6275(7)	7781(10)	64(4)
C16	4141(15)	6594(6)	880(8)	45(2)
O1	5978(8)	5015(5)	968(5)	40.0(15)
O2	4631(8)	3335(4)	3232(5)	36.3(14)
O3	4842(12)	2369(2)	6829(5)	60(2)
O4	5086(12)	5850(5)	4150(6)	52.6(19)
O5	3908(8)	5921(4)	1650(5)	38.0(14)
O6	7166(10)	5776(5)	6175(6)	55.0(19)
O7	4810(11)	5551(5)	7069(5)	51.2(19)
C1″	10408(15)	4450(8)	5561(8)	51(3)
C2′	9824(13)	4211(6)	4413(7)	35(2)
C3′	10026(12)	4927(7)	3622(8)	41(2)
C4′	9954(13)	4633(6)	2480(7)	33(2)
C5′	9909(12)	5071(5)	657(7)	33(2)
C6′	10017(13)	5755(5)	7(6)	34(2)
C7′	10195(14)	5625(7)	−1081(9)	46(2)
C8′	10290(14)	4774(2)	−1439(8)	41(2)
C9′	10157(12)	4078(5)	−748(7)	33.0(19)
C10′	9998(12)	4195(5)	358(8)	33(2)
C11′	9800(12)	3506(6)	1119(8)	37(2)
C12′	9925(12)	3776(7)	2232(9)	41(2)
C13′	10166(13)	3105(7)	3112(7)	38(2)
C14′	10411(13)	3196(6)	−1251(7)	37(2)
C15′	9430(19)	2284(6)	−2704(8)	55(3)
C16′	8710(15)	2010(7)	3960(8)	49(2)
O1′	10839(9)	3486(5)	4109(5)	43.1(15)
O2′	9821(9)	5238(4)	1751(5)	37.1(15)
O3′	10415(11)	6243(2)	−1778(5)	54(2)
O4′	9534(10)	2760(5)	783(5)	44.6(17)
O5′	11626(10)	2728(5)	−972(6)	52.3(18)
O6′	9196(10)	3054(5)	−2069(6)	51.3(19)
O7′	8541(8)	2709(5)	3171(5)	42.2(15)

Table S3. Anisotropic displacement parameters (Å^2 × 10^3) for phomopsichin A (**1**). The anisotropic displacement factor exponent takes the form: $-2\pi^2[h^2a^{*2}U_{11} + 2hka^*b^*U_{12} + \ldots]$.

Atom	U_{11}	U_{22}	U_{33}	U_{23}	U_{13}	U_{12}
C1	61(6)	32(5)	29(5)	−3(4)	8(4)	−10(4)
C2	54(5)	15(4)	30(4)	−9(3)	7(4)	−11(4)
C3	54(6)	16(4)	40(5)	−4(4)	5(4)	−5(4)
C4	32(5)	22(4)	57(6)	−15(4)	6(4)	−4(4)
C5	37(5)	28(5)	36(5)	−7(4)	3(3)	8(4)
C6	57(6)	23(5)	40(5)	2(4)	7(4)	8(4)
C7	68(7)	21(5)	44(6)	16(4)	15(5)	5(4)

C8	67(7)	32(5)	33(5)	3(4)	9(4)	5(4)
C9	38(5)	25(4)	41(5)	2(4)	2(4)	9(4)
C10	43(5)	12(4)	36(5)	4(3)	4(4)	1(3)
C11	58(6)	27(5)	30(5)	−4(4)	3(4)	−3(4)
C12	64(6)	11(4)	20(4)	1(3)	0(4)	−2(4)
C13	44(5)	23(4)	35(5)	0(4)	4(4)	−6(4)
C14	52(6)	31(5)	36(5)	−1(4)	8(4)	3(4)
C15	123(11)	22(5)	47(6)	−4(5)	9(7)	3(6)
C16	67(7)	22(4)	46(5)	4(4)	2(5)	0(4)
O1	41(4)	28(3)	50(4)	−9(3)	4(3)	−4(3)
O2	54(4)	19(3)	36(3)	−10(2)	7(3)	2(3)
O3	114(7)	24(3)	43(4)	11(3)	14(4)	2(4)
O4	104(6)	21(3)	42(4)	−7(3)	6(4)	1(3)
O5	52(4)	18(3)	44(3)	−2(3)	7(3)	1(3)
O6	66(5)	38(4)	62(5)	−7(3)	11(4)	−15(4)
O7	96(6)	25(3)	34(4)	−7(3)	19(4)	−1(3)
C1′	60(7)	47(6)	47(6)	−18(5)	5(5)	−3(5)
C2′	48(5)	24(4)	35(5)	5(4)	10(4)	7(4)
C3′	31(5)	41(5)	52(6)	−19(5)	11(4)	−6(4)
C4′	55(5)	22(4)	24(4)	15(3)	8(4)	5(4)
C5′	49(5)	9(4)	41(5)	−5(4)	7(4)	0(3)
C6′	50(6)	11(4)	33(5)	4(3)	6(4)	3(3)
C7′	52(6)	27(5)	60(7)	1(4)	12(5)	−2(4)
C8′	63(6)	21(4)	40(5)	8(4)	13(4)	3(4)
C9′	45(5)	9(4)	45(5)	3(4)	5(4)	−2(3)
C10′	50(5)	10(4)	44(5)	4(3)	5(4)	−2(3)
C11′	49(5)	23(5)	38(5)	16(4)	5(4)	−5(4)
C12′	32(5)	33(5)	57(6)	−13(5)	3(4)	−6(4)
C13′	61(6)	28(5)	24(4)	3(4)	5(4)	15(4)
C14′	47(5)	26(5)	38(5)	5(4)	11(4)	0(4)
C15′	116(10)	18(4)	30(5)	−1(4)	3(5)	3(5)
C16′	66(7)	34(5)	46(6)	12(4)	7(5)	−3(5)
O1′	56(4)	38(4)	36(3)	−3(3)	8(3)	5(3)
O2′	57(4)	14(3)	40(4)	2(2)	4(3)	1(2)
O3′	104(6)	22(3)	38(4)	10(3)	12(4)	−3(3)
O4′	83(5)	20(3)	32(3)	−5(3)	13(3)	−9(3)
O5′	66(5)	26(3)	66(5)	−7(3)	11(4)	2(3)
O6′	76(5)	23(3)	53(4)	−8(3)	−4(4)	5(3)
O7′	42(4)	29(3)	56(4)	0(3)	7(3)	−3(3)

Table S4. Bond lengths for phomopsichin A (**1**).

Atom	Atom	Length/Å	Atom	Atom	Length/Å
C1	C2	1.517(12)	C1′	C2′	1.501(13)
C2	O1	1.453(10)	C2′	O1′	1.442(11)
C2	C3	1.480(12)	C2′	C3′	1.506(13)
C3	C4	1.489(12)	C3′	C4′	1.494(12)
C4	O2	1.354(12)	C4′	O2′	1.305(9)
C4	C12	1.362(11)	C4′	C12′	1.367(12)
C5	O2	1.360(10)	C5′	C6′	1.345(10)
C5	C10	1.403(12)	C5′	O2′	1.400(10)
C5	C6	1.407(11)	C5′	C10′	1.415(11)

C6	C7	1.363(13)	C6′	C7′	1.395(13)
C7	O3	1.332(10)	C7′	O3′	1.320(11)
C7	C8	1.382(12)	C7′	C8′	1.400(11)
C8	C9	1.384(11)	C8′	C9′	1.395(10)
C9	C10	1.421(12)	C9′	C10′	1.411(13)
C9	C14	1.490(13)	C9′	C14′	1.528(12)
C10	C11	1.443(12)	C10′	C11′	1.451(11)
C11	O4	1.286(11)	C11′	O4′	1.242(11)
C11	C12	1.463(12)	C11′	C12′	1.446(14)
C12	C13	1.463(12)	C12′	C13′	1.511(13)
C13	O5	1.422(11)	C13′	O7′	1.392(12)
C13	O1	1.444(11)	C13′	O1′	1.423(11)
C14	O6	1.210(12)	C14′	O5′	1.200(12)
C14	O7	1.354(11)	C14′	O6′	1.321(12)
C15	O7	1.458(13)	C15′	O6′	1.457(11)
C16	O5	1.446(11)	C16′	O7′	1.464(11)

Table S5. Bond angles for phomopsichin A (**1**).

Atom	Atom	Atom	Angle/°	Atom	Atom	Atom	Angle/°
O1	C2	C3	111.7(7)	O1′	C2′	C1′	109.4(8)
O1	C2	C1	104.9(7)	O1′	C2′	C3′	108.0(7)
C3	C2	C1	115.4(7)	C1′	C2′	C3′	113.7(9)
C2	C3	C4	109.8(7)	C4′	C3′	C2′	113.8(8)
O2	C4	C12	124.4(8)	O2′	C4′	C12′	123.2(8)
O2	C4	C3	112.2(7)	O2′	C4′	C3′	115.9(8)
C12	C4	C3	123.4(9)	C12′	C4′	C3′	120.8(8)
O2	C5	C10	123.7(8)	C6′	C5′	O2′	117.0(7)
O2	C5	C6	115.5(8)	C6′	C5′	C10′	126.4(8)
C10	C5	C6	120.8(8)	O2′	C5′	C10′	116.5(7)
C7	C6	C5	120.1(8)	C5′	C6′	C7′	119.4(8)
O3	C7	C6	124.3(8)	O3′	C7′	C6′	124.7(8)
O3	C7	C8	115.6(9)	O3′	C7′	C8′	117.5(9)
C6	C7	C8	120.1(8)	C6′	C7′	C8′	117.5(9)
C7	C8	C9	121.5(9)	C9′	C8′	C7′	121.7(9)
C8	C9	C10	119.6(8)	C8′	C9′	C10′	121.7(7)
C8	C9	C14	118.7(8)	C8′	C9′	C14′	114.8(8)
C10	C9	C14	121.3(8)	C10′	C9′	C14′	123.1(7)
C5	C10	C9	117.9(8)	C9′	C10′	C5′	113.2(7)
C5	C10	C11	116.5(8)	C9′	C10′	C11′	124.9(7)
C9	C10	C11	125.6(8)	C5′	C10′	C11′	121.7(9)
O4	C11	C10	121.3(8)	O4′	C11′	C12′	125.9(8)
O4	C11	C12	120.1(8)	O4′	C11′	C10′	119.6(9)
C10	C11	C12	118.6(8)	C12′	C11′	C10′	114.5(8)
C4	C12	C11	117.4(8)	C4′	C12′	C11′	119.9(9)
C4	C12	C13	120.4(8)	C4′	C12′	C13′	120.7(9)
C11	C12	C13	122.1(7)	C11′	C12′	C13′	119.3(8)
O5	C13	O1	109.7(7)	O7′	C13′	O1′	112.1(7)
O5	C13	C12	108.2(7)	O7′	C13′	C12′	107.7(7)
O1	C13	C12	112.5(7)	O1′	C13′	C12′	110.7(8)
O6	C14	O7	122.0(9)	O5′	C14′	O6′	125.2(9)
O6	C14	C9	128.0(9)	O5′	C14′	C9′	123.5(9)

O7	C14	C9	109.3(8)	O6′	C14′	C9′	111.1(8)
C13	O1	C2	110.7(7)	C13′	O1′	C2′	113.7(7)
C4	O2	C5	118.6(7)	C4′	O2′	C5′	122.5(7)
C13	O5	C16	113.0(7)	C14′	O6′	C15′	116.3(8)
C14	O7	C15	118.1(9)	C13′	O7′	C16′	110.5(7)

Table S6. Torsion angles for phomopsichin A (**1**).

A	B	C	D	Angle/°	A	B	C	D	Angle/°
O1	C2	C3	C4	45.0(10)	C1′	C2′	C3′	C4′	163.3(8)
C1	C2	C3	C4	164.8(8)	C2′	C3′	C4′	O2′	168.8(8)
C2	C3	C4	O2	170.8(8)	C2′	C3′	C4′	C12′	−7.5(13)
C2	C3	C4	C12	−12.1(13)	O2′	C5′	C6′	C7′	176.9(8)
O2	C5	C6	C7	179.5(9)	C10′	C5′	C6′	C7′	1.6(15)
C10	C5	C6	C7	−0.8(13)	C5′	C6′	C7′	O3′	−175.9(10)
C5	C6	C7	O3	179.6(9)	C5′	C6′	C7′	C8′	−1.3(14)
C5	C6	C7	C8	0.7(15)	O3′	C7′	C8′	C9′	177.0(9)
O3	C7	C8	C9	−179.1(9)	C6′	C7′	C8′	C9′	2.0(15)
C6	C7	C8	C9	0.0(16)	C7′	C8′	C9′	C10′	−2.9(15)
C7	C8	C9	C10	−0.4(15)	C7′	C8′	C9′	C14′	−176.1(9)
C7	C8	C9	C14	−173.3(10)	C8′	C9′	C10′	C5′	2.7(13)
O2	C5	C10	C9	−180.0(8)	C14′	C9′	C10′	C5′	175.4(8)
C6	C5	C10	C9	0.4(13)	C8′	C9′	C10′	C11′	178.0(9)
O2	C5	C10	C11	3.1(13)	C14′	C9′	C10′	C11′	−9.2(14)
C6	C5	C10	C11	−176.6(8)	C6′	C5′	C10′	C9′	−2.2(14)
C8	C9	C10	C5	0.2(13)	O2′	C5′	C10′	C9′	−177.5(7)
C14	C9	C10	C5	172.9(8)	C6′	C5′	C10′	C11′	−177.7(9)
C8	C9	C10	C11	176.9(9)	O2′	C5′	C10′	C11′	7.0(13)
C14	C9	C10	C11	−10.4(13)	C9′	C10′	C11′	O4′	−9.3(14)
C5	C10	C11	O4	167.4(9)	C5′	C10′	C11′	O4′	165.7(9)
C9	C10	C11	O4	−9.3(15)	C9′	C10′	C11′	C12′	171.3(8)
C5	C10	C11	C12	−9.7(12)	C5′	C10′	C11′	C12′	−13.8(13)
C9	C10	C11	C12	173.6(8)	O2′	C4′	C12′	C11′	2.1(14)
O2	C4	C12	C11	−1.5(14)	C3′	C4′	C12′	C11′	178.1(8)
C3	C4	C12	C11	−178.3(8)	O2′	C4′	C12′	C13′	177.3(9)
O2	C4	C12	C13	174.8(8)	C3′	C4′	C12′	C13′	−6.7(14)
C3	C4	C12	C13	−2.0(14)	O4′	C11′	C12′	C4′	−170.1(10)
O4	C11	C12	C4	−168.1(9)	C10′	C11′	C12′	C4′	9.4(13)
C10	C11	C12	C4	9.1(13)	O4′	C11′	C12′	C13′	14.7(14)
O4	C11	C12	C13	15.7(14)	C10′	C11′	C12′	C13′	−165.9(8)
C10	C11	C12	C13	−167.2(9)	C4′	C12′	C13′	O7′	108.2(10)
C4	C12	C13	O5	104.5(10)	C11′	C12′	C13′	O7′	−76.6(10)
C11	C12	C13	O5	−79.3(10)	C4′	C12′	C13′	O1′	−14.7(12)
C4	C12	C13	O1	−16.7(12)	C11′	C12′	C13′	O1′	160.5(8)
C11	C12	C13	O1	159.4(8)	C8′	C9′	C14′	O5′	114.4(10)
C8	C9	C14	O6	116.6(12)	C10′	C9′	C14′	O5′	−58.7(13)
C10	C9	C14	O6	−56.1(14)	C8′	C9′	C14′	O6′	−61.3(11)
C8	C9	C14	O7	−53.5(11)	C10′	C9′	C14′	O6′	125.6(9)
C10	C9	C14	O7	133.7(9)	O7′	C13′	O1′	C2′	−67.5(9)
O5	C13	O1	C2	−70.7(9)	C12′	C13′	O1′	C2′	52.8(10)
C12	C13	O1	C2	49.7(10)	C1′	C2′	O1′	C13′	168.6(8)
C3	C2	O1	C13	−66.3(9)	C3′	C2′	O1′	C13′	−67.1(10)

C1	C2	O1	C13	167.9(7)	C12′	C4′	O2′	C5′	−10.1(14)
C12	C4	O2	C5	−5.3(13)	C3′	C4′	O2′	C5′	173.7(8)
C3	C4	O2	C5	171.8(7)	C6′	C5′	O2′	C4′	−170.5(9)
C10	C5	O2	C4	4.4(12)	C10′	C5′	O2′	C4′	5.3(12)
C6	C5	O2	C4	−175.9(8)	C6′	C7′	O3′	H3′	15.6
O1	C13	O5	C16	−60.7(9)	C8′	C7′	O3′	H3′	−158.9
C12	C13	O5	C16	176.3(7)	O5′	C14′	O6′	C15′	−3.4(14)
O6	C14	O7	C15	−2.3(14)	C9′	C14′	O6′	C15′	172.2(8)
C9	C14	O7	C15	168.6(8)	O1′	C13′	O7′	C16′	−61.8(9)
O1′	C2′	C3′	C4′	41.6(10)	C12′	C13′	O7′	C16′	176.1(8)

Table S7. Hydrogen atom coordinates (Å × 10^4) and isotropic displacement parameters (Å^2 × 10^3) for phomopsichin A (**1**).

Atom	*x*	*y*	*z*	U(eq)
H1A	5109	4611	−967	61
H1B	4375	3658	−820	61
H1C	6407	3872	−459	61
H2	3529	4578	537	40
H3A	5893	3268	1337	44
H3B	3778	3238	1223	44
H6	4508	2282	4735	48
H8	5382	3892	7253	53
H13	6432	5819	2222	41
H15A	6399	6138	8236	96
H15B	4362	6386	8237	96
H15C	5487	6787	7348	96
H16A	5258	6894	1088	68
H16B	3163	7004	867	68
H16C	4161	6341	162	68
H3	4648	1843	6718	90
H1′1	9922	4037	6043	77
H1′2	9983	5029	5705	77
H1′3	11699	4439	5683	77
H2′	8553	4041	4356	42
H3′1	9079	5355	3679	49
H3′2	11169	5219	3823	49
H6′	9972	6322	287	41
H8′	10450	4668	−2171	49
H13′	11026	2664	2911	45
H15D	10688	2201	−2770	83
H15E	8788	2351	−3423	83
H15F	8972	1783	−2348	83
H16D	9736	1655	3847	73
H16E	7642	1654	3874	73
H16F	8864	2252	4689	73
H3′	10710	6751	−1617	81

Refinement model description number of restraints—0, number of constraints—unknown, details: N/A. This report has been created with Olex2, compiled on 19.02.2016 svn.r3266 for OlexSys. Please let us know if there are any errors or if you would like to have.

Table S8. Inhibitory activities against AchE as well as α-glucosidase, and the radical scavenging effects on DPPH as well as OH of compounds **1**–**5**.

	**1**	**2**	**3**	**4**	**5** [e]	**Positive Control**
	**Inhibitory Rate (%)**					**IC_{50}**
AchE (250 μm)	38.4	2.7	9.1	11.3	/	45.2 nM [a]
α-glucosidase (250 μmol)	/	12.5	21.3	15.7	38.9	29.5 μM [b]
DPPH (1 mM)	18.0	25.3	52.0 (IC_{50} 0.86 mM)	17.0	40.0	25.8 μM [c]
OH (1 mM)	3.5	67.6 (IC_{50} 0.62 mM)	53.0 (IC_{50} 0.49 mM)	23.6	/	35.7 μM [d]

[a]: huperzine A; [b]: *trans*-resveratrol; [c]: 2,6-ditertbutyl-4-methylphenol; [d]: vitamin C; [e]: The concentration of compound **5** was half of the labeled concentration.

Table S9. Antimicrobial activity of compounds **1**–**5**.

	**1**	**2**	**3**	**4**	**5**
	**Inhibitory Rates (%)**				
	**100 μg/mL**				
Escherichia coli	1.3	/	15.3	8.9	48.4
Escherichia coli	0.5	/	10.4	0.4	34.5
Citrobacter freundii	32.6	41.7	41.1	34.3	26.1
Sarcina lutea	7.3	3.1	7.1	4.2	/
Staphylococcus albus Rosenbach	34.1	42.5	39.3	41.3	/
Salmonella enterica subsp. *Enterica*	14.0	18.7	11.6	18.3	/
Staphylococcus aureus	18.0	16.4	17.4	27.6	24.5
Vibrio parahaemolyticus	40.3	33.4	43.6	36.6	2.5
Vibrio parahaemolyticus	50.2	51.6	54.4	59.6	/
Bacillus cereus Flankland	39.7	38.7	29.1	39.4	34.8
Acinetobacter	17.3	23.9	24.7	37.8	/
Bacillus subtilis	14.8	18.0	16.6	15.0	3.5
Vibrio harvey	17.2	15.7	15.5	14.7	39.5
